# Single‐Cell Analysis Clarifies Pathological Heterogeneity in Tenosynovial Giant Cell Tumor and Identifies Biomarkers for Predicting Disease Recurrence

**DOI:** 10.1002/advs.202415835

**Published:** 2025-03-24

**Authors:** Yubin Xie, Chen Chen, Fei Wu, Yiman Peng, Jing Su, Yang Zhao, Hongjie Huang, Zhong Alan Li, Yin Pei, Wencui Li, Yi He, Tianchen Xue, Chenxi Cao, Sui Peng, Xin Zhang, Weidong Song

**Affiliations:** ^1^ Institute of Precision Medicine The First Affiliated Hospital Sun Yat‐sen University Guangzhou Guangdong 518000 P. R. China; ^2^ Trauma Orthopedics Foot and Ankle Surgery Sun Yat‐Sen Memorial Hospital Sun Yat‐Sen University 107 Yanjiangxi Road Guangzhou Guangdong 510120 P. R. China; ^3^ Department of Sports Medicine Peking University Third Hospital 49 North Garden Road, Haidian District Beijing 100191 P. R. China; ^4^ Department of Pathology School of Basic Medical Sciences Peking University Third Hospital Peking University Health Science Center Beijing 100191 P. R. China; ^5^ Department of Laboratory Medicine Peking University Third Hospital 49 North Garden Road, Haidian District Beijing 100191 P. R. China; ^6^ Department of Biomedical Engineering The Chinese University of Hong Kong Hong Kong SAR 999077 China; ^7^ Hand and Foot Surgery Department The First Affiliated Hospital of Shenzhen University Shenzhen Second People's Hospital Shenzhen Guangdong 518035 P. R. China; ^8^ Emergency Trauma Center Fifth Affiliated Hospital of Guangzhou Medical University Guangzhou Guangdong 510700 P. R. China; ^9^ Department of Pathology The First Affiliated Hospital Sun Yat‐sen University Guangzhou Guangdong 518000 P. R. China; ^10^ Department of Gastroenterology and Hepatology Sun Yat‐sen University First Affiliated Hospital Guangzhou Guangdong 518000 P. R. China; ^11^ Clinical Trials Unit The First Affiliated Hospital Sun Yat‐sen University Guangzhou 518000 P. R. China

**Keywords:** local aggression, recurrence, single‐cell RNA sequencing, tenosynovial giant cell tumors

## Abstract

Diffuse‐type tenosynovial giant cell tumor (D‐TGCT) and localized‐type tenosynovial giant cell tumor (L‐TGCT) share common genomic aberrations and histopathological features, but the former has a more aggressive nature and a higher recurrence rate, leading to worse prognoses for patients. In this study, single‐cell RNA sequencing (scRNA‐seq) on human D‐TGCT and L‐TGCT lesions is conducted to discover transcriptional differences. A unique cluster of tumor cells in D‐TGCT is identified that regulated differentiation of *CD34* ^+^ fibroblasts into *MMP*3^+^ fibroblasts or *APOE* ^+^ fibroblasts via COL6A3 − (ITGAV + ITGB8) interaction. The *APOE* ^+^ fibroblasts further activated *IL‐1B* ^+^ *CCL20* ^+^ macrophages through the CXCL12/CXCR4 axis. *IL‐1B* ^+^ *CCL20* ^+^ macrophages and *MMP3* ^+^ fibroblasts participated in local aggression of D‐TGCT. Two effective biomarkers, *ROR1* and *PRKD1* are also identified and validated, to predict disease recurrence. This study not only clarified the underlying mechanisms of aggressive behavior in D‐TGCT but also provided a theoretical basis and potential targets for intervention into and treatment of this disease.

## Graphical abstract

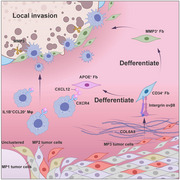

## 1 Introduction

Tenosynovial giant cell tumor (TGCT), also known as pigmented villonodular synovitis, is a rare proliferative mesenchymal neoplasm that affects bones, synovium, tendon sheaths, and bursae of the joints.^[^ ^1^ ^]^ Two distinct subtypes, L‐TGCT and D‐TGCT, are defined by different radiological, biological, and clinical features.^[^ ^2^, ^3^ ^]^ L‐TGCT typically manifests as a well‐circumscribed, non‐destructive nodule, whereas D‐TGCT presents as uncontrolled synovial proliferation with locally aggressive and invasive behavior, causing severe joint pain and recurrent articular hemorrhage. Extra‐articular infiltration and malignant transformation have been incidentally reported,^[^ ^4^, ^5^ ^]^ forcing amputation of limbs and leaving patients disabled.^[^ ^6^, ^7^ ^]^

Currently, no recognized clinical guideline for the treatment of TGCT exists, but the mainstay treatment option is still surgical resection, whether open or arthroscopic, to relieve pain and lower the risk of joint destruction.^[^ ^8^ ^]^ For L‐TGCT, which often occurs in the hands and feet, surgical excision with clear margins is an effective treatment; the average recurrence rate after these procedures is <6%.^[^ ^9^ ^]^ However, D‐TGCT has a much higher recurrence rate, almost 44%, after surgical resection, which seriously affects patients’ quality of life (QoL)^[^ ^10^, ^11^ ^]^ and necessitates additional interventions such as radiotherapy^[^ ^12^, ^13^ ^]^ and drug therapy.^[^ ^14^ ^]^ The long‐term risks of vascular necrosis, joint fibrosis, and stiffness caused by radiation therapy, as well as other sequelae, merit further research still.^[^ ^15^ ^]^ In addition, drug research mainly focuses on colony‐stimulating factor 1 receptor (*CSF1R*) inhibitors, such as pexidartinib, the first drug to receive US Food and Drug Administration (FDA) approval for treating D‐TGCT.^[^ ^14^ ^]^ However, the poor effectiveness and side effects of these medications, such as liver and kidney toxicity, remain unresolved challenges.^[^ ^16^ ^]^ Therefore, comprehensive treatment of D‐TGCT is difficult to achieve.

To date, the pathogenesis and molecular features of TGCT are still unclear and debated, with conflicting theories proposing it as either an inflammatory disease or a neoplastic disease. This lack of clarity restricts the development of systemic treatment strategies for TGCT, especially molecular targeted therapy and drug research. Previous studies involving pathological and flow cytometric (FCM) analyses have demonstrated that both subtypes of TGCT are characterized by disruption and rearrangement of the 3′‐end of CSF^[^ ^17^, ^18^, ^19^ ^]^ and that both are mainly composed of mononuclear macrophages, fibroblasts, and lymphocytes.^[^ ^20^, ^21^ ^]^ However, the clinical characteristics, postoperative‐recurrence rates, and prognoses of the two subtypes are quite different. Why D‐TGCT is more aggressive and has a higher recurrence rate is not fully understood. Given these limitations, revealing the mechanisms that drive such aggression and recurrence has become crucial for developing novel effective treatments and early‐intervention strategies for D‐TGCT.

Interactions among tumor cells, immune cells, and stromal cells in the tumor microenvironment (TME) could influence tumor progression and immune status.^[^ ^22^, ^23^, ^24^, ^25^ ^]^ However, the differences in TME between the two TGCT subtypes, the classification and functional annotation of each cell subpopulation, and potential biomarkers for predicting recurrence have not been explored. The advent of scRNA‐seq provides an optimal means of shedding light on the pathological profile and molecular features of TGCT. In this study, we examined 10 D‐TGCT samples and 7 L‐TGCT samples using scRNA‐seq to investigate intratumoral heterogeneity and analyze the TME as well as intercellular interactions to explain the discrepancy in manifestations between D‐TGCT and L‐TGCT. Since osteoarthritis (OA) is a degenerative disease of articular cartilage, characterized by relatively mild synovial inflammation compared to other arthritis such as rheumatoid arthritis (RA), we also sequenced 3 OA samples as control in our study. Using in silico analysis, we identified and validated two effective biomarkers to predict disease recurrence. Our findings yielded an in‐depth single‐cell transcriptomic atlas of TGCT and can offer a theoretical basis of and potential targets for intervention into and treatment of this disease.

## 2 Results

### 2.1 Single‐Cell Profiling of D‐TGCT and L‐TGCT

**Figure 1:**
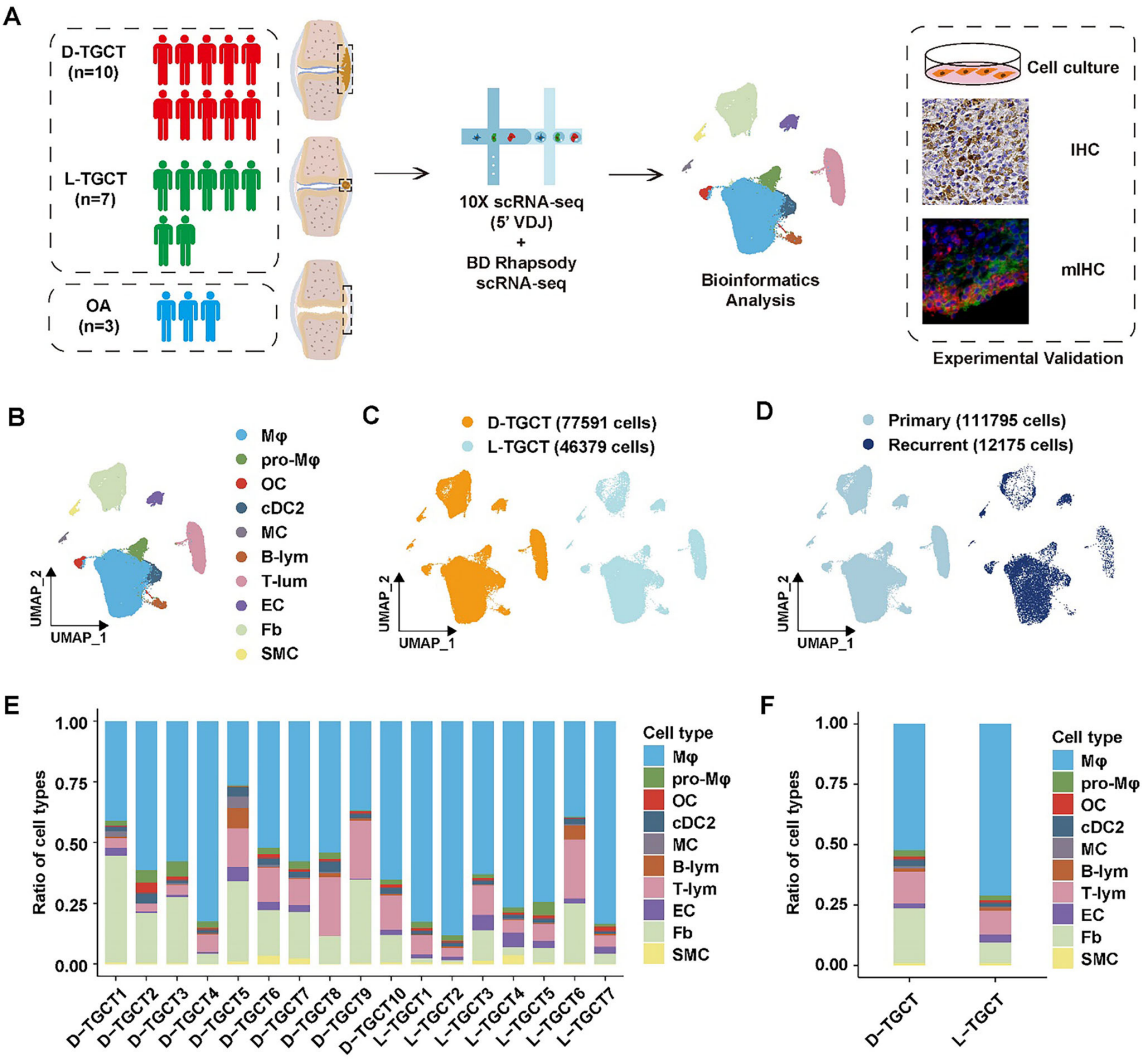
Single‐cell transcriptome atlas of diffused‐type tenosynovial giant cell tumor (D‐TGCT) and localized‐type tenosynovial giant cell tumor (L‐TGCT). A) Workflow of the current study. B) UMAP projection of 123970 cells from 10 D‐TGCT and 7 L‐TGCT samples, which were annotated into 10 main cell types. Each dot represents a single cell and is colored according to its cell population. C) UMAP projection of all TGCT cells grouped by different subtypes. D) UMAP projection of all TGCT cells grouped by disease status. E) Histogram showing the ratio of each cell type across the 10 D‐TGCT and 7 L‐TGCT samples. F) Histogram showing the ratio of each cell type between D‐TGCT and L‐TGCT samples.

To fully decipher global differences in TMEs between D‐TGCT and L‐TGCT, we performed scRNA‐seq on 17 samples (nine primary D‐TGCT, one recurrent D‐TGCT, six primary L‐TGCT, and one recurrent L‐TGCT) as a discovery cohort using the 10x Genomics Chromium platform (10x Genomics, Inc., Pleasanton, CA, USA; **Figure** **1**A). Following standard data processing and quality control (QC) procedures, we obtained 123970 TGCT cells, which were then clustered into 32 subpopulations via uniform manifold approximation and projection (UMAP; Figure S1A, Supporting Information). The uniform distribution of these 32 cell populations across each sample suggested successful correction for the batch effect (Figure S1B, Supporting Information). Then, we annotated these populations into 10 major cell types based on expression levels of canonical marker genes (Figure 1B; Figure S1C, Supporting Information), including macrophages (Mφs), proliferating macrophages (pro‐Mφs), osteoclasts (OCs), type 2 conventional dendritic cells (cDC2s), mast cells (MCs), B lymphocytes (B‐lyms), T lymphocytes (T‐lyms), endothelial cells (ECs), fibroblasts (Fbs), and smooth‐muscle cells (SMCs). All annotated cell types were distributed consistently between D‐TGCT and L‐TGCT (Figure 1C), as well as between recurrent and primary TGCT (Figure 1D). Similarly, we clustered and annotated five major cell types in OA synovium (Figure S1D,E, Supporting Information): Mφs, T‐lyms, MCs, Fbs, and ECs. Compared with OA synovium, immune cells were the predominant cellular components in TGCT, suggesting a significant pathological role of these cells in the disease.

Notably, scRNA‐seq analysis identified macrophages and fibroblasts as the predominant cellular components (Figure 1E,F), exhibiting differential abundance between D‐TGCT and L‐TGCT (Figure S1F, Supporting Information). Mφs have previously been reported as the responsive cell type in TGCT.^[^ ^17^, ^26^ ^]^ Therefore, we first focused on these two cell types and their heterogeneity between D‐TGCT and L‐TGCT.

### 2.2 *IL‐1B* ^+^ *CCL20* ^+^ Macrophages in D‐TGCT Showed Increased Invasive Capability, Potentially Modulated by Fibroblasts

**Figure 2:**
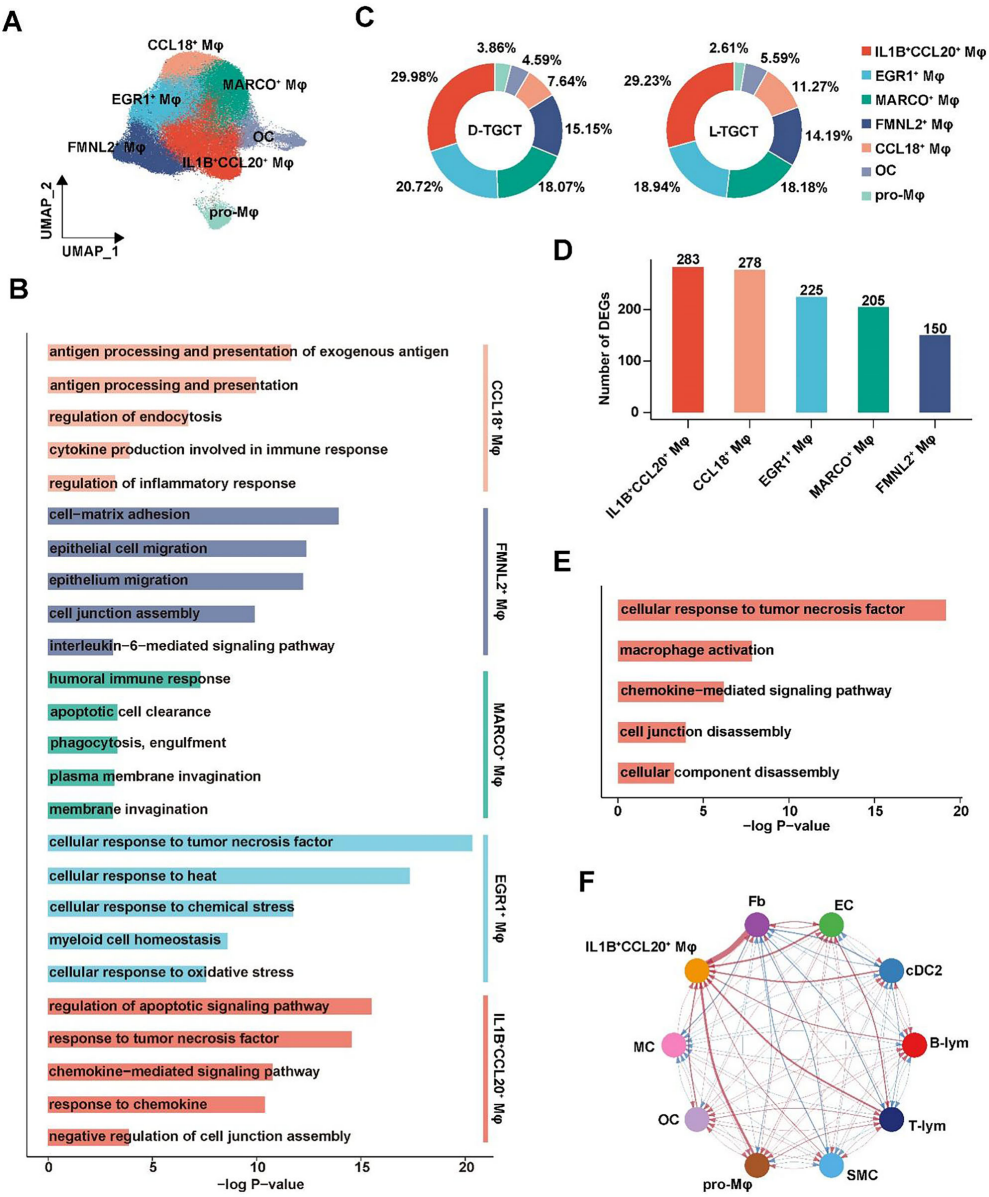
Single‐cell transcriptome atlas of macrophages. A) UMAP projection of seven subpopulations generated from unsupervised clustering of macrophagic lineages. B) GO enrichment analysis of DEGs of each Mφ subpopulation, suggesting that different biological functions were present in different Mφ subpopulations. C) Pie charts showing the proportion of each Mφ subpopulation in primary TGCT. D) Histogram showing the number of DEGs of each Mφ subpopulation between D‐TGCT and L‐TGCT. E) Bar plot showing the enriched GO pathways in *IL‐1B* ^+^ *CCL20* ^+^ Mφs from D‐TGCT compared with those from L‐TGCT. F) Circle plot showing the cell–cell communication between *IL‐1B* ^+^ *CCL20* ^+^ Mφs and other major immune cell populations. Line thickness indicates relative interaction intensity. Red and blue represent higher interaction intensities observed in D‐TGCT and L‐TGCT, respectively.

Macrophagic lineages from primary TGCT, including Mφs, pro‐Mφs, and OCs, were clustered into seven subpopulations (**Figure** **2**A) and subsequently annotated as *IL‐1B* ^+^ *CCL20* ^+^ Mφs, *EGR1* ^+^ Mφs, *MARCO* ^+^ Mφs, *FMNL2* ^+^ Mφs, *CCL18* ^+^ Mφs, pro‐Mφs, and OCs based on their differentially expressed genes (DEGs; Figure S2A, Supporting Information). We then conducted Gene Ontology (GO) functional‐enrichment analysis of these DEGs to infer their cellular functions. GO analysis revealed that upregulated DEGs in *IL‐1B* ^+^ *CCL20* ^+^ Mφs were enriched in pathways pertinent to inflammation activation, M1 polarization, and invasion (Figure 2B). In particular, “regulation of apoptotic signaling pathway,” “response to tumor necrosis factor,” “chemokine‐mediated signaling pathway,” and “negative regulation of cell junction assembly” were enriched in *IL‐1B* ^+^ *CCL20* ^+^ Mφs. Similarly, we designated the remaining Mφ subpopulations based on their respective enriched GO terms: *EGR1* ^+^ Mφs were termed another immune‐activated subset due to enrichment of “cellular response to tumor necrosis factor,” “cellular response to heat,” and “cellular response to chemical stress”; *MARCO* ^+^ Mφs were termed phagocytic type due to enrichment of “humoral immune response,” “apoptotic‐cell clearance,” and terms related to phagocytosis; *FMNL2* ^+^ Mφs were subsets related to angiogenesis; and *CCL18* ^+^ Mφs were subsets related to inherent immunity (Figure 2B). We further validated the functional enrichment analysis results by calculating phenotypic gene set scores for each Mφ subset. Consistently, the expression levels of M1, pro‐inflammatory, and Mφ invasion signatures of *IL‐1B* ^+^ *CCL20* ^+^ Mφs were higher than those of the other Mφs (Figure S2B, Supporting Information). Based on these findings, we assumed that *IL‐1B* ^+^ *CCL20* ^+^ Mφs might contribute to the aggressive behavior of D‐TGCT.

Notably, *IL‐1B* ^+^ *CCL20* ^+^ Mφs constituted the majority subpopulation of Mφs, but they showed minimal variation between D‐TGCT and L‐TGCT with no statistically significant differences (Figure 2C; Figure S2C, Supporting Information). We also performed diffusion map dimensionality reduction to interrogate differentiation trajectories of macrophage subpopulations across two types of TGCT. As mentioned above, macrophages in TGCT originated from circulating monocytes^[^ ^17^ ^]^. We accordingly calculated the monocyte signature score for each macrophage subpopulation to identify a monocyte‐like subset and found out *MARCO* ^+^ Mφs exhibited the highest score (Figure S2D, Supporting Information). By utilizing it as the starting point, we constructed the differentiation trajectories of macrophage subpopulations (Figure S2E,F, Supporting Information). RNA velocity showed a similar developmental trajectory, starting with *MARCO* ^+^ Mφs and ending with *FMNL2^+^* Mφs (Figure S2G, Supporting Information). Consistent with the results of cellular proportion analysis, the developmental trajectories of all macrophage subsets exhibited no differences between D‐TGCT and L‐TGCT (Figure S2H, Supporting Information), prompting us to further investigate whether they exhibited discrepancies in functionality.

To identify the functional variances of Mφ subpopulations between D‐TGCT and L‐TGCT, we calculated the number of DEGs within each Mφ lineage across both types of TGCT. Of these lineages, *IL‐1B* ^+^ *CCL20* ^+^ Mφs had the highest DEG count, suggesting the most pronounced functional diversity (Figure 2D). We next conducted GO functional enrichment analysis of DEGs of *IL‐1B* ^+^ *CCL20* ^+^ Mφs from D‐TGCT compared with those from L‐TGCT. We found that the upregulated genes in D‐TGCT were enriched in “cellular response to tumor necrosis factor,” “macrophage activation,” “chemokine‐mediated signaling pathway,” “cell junction disassembly,” and “cellular component disassembly” (Figure 2E), indicating that *IL‐1B* ^+^ *CCL20* ^+^ Mφs from D‐TGCT had a more apparently invasive phenotype than those from L‐TGCT. To further investigate potential upstream cell populations contributing to the altered functions of *IL‐1B* ^+^ *CCL20* ^+^ Mφs across both types of TGCT, we conducted cell–cell interaction (CCI) analysis using the R software package CellChat. Extensive cellular communication was observed in TGCT, and it was stronger between Fbs and *IL‐1B* ^+^ *CCL20* ^+^ Mφs in D‐TGCT than in L‐TGCT (Figure 2F). This suggested that the functional variance of *IL‐1B* ^+^ *CCL20* ^+^ Mφs in D‐TGCT might be influenced by Fbs.

### 2.3 Higher Proportions of *MMP3* ^+^ Fb and *APOE* ^+^ Fb are Associated with the Aggressive Behavior of D‐TGCT

**Figure 3:**
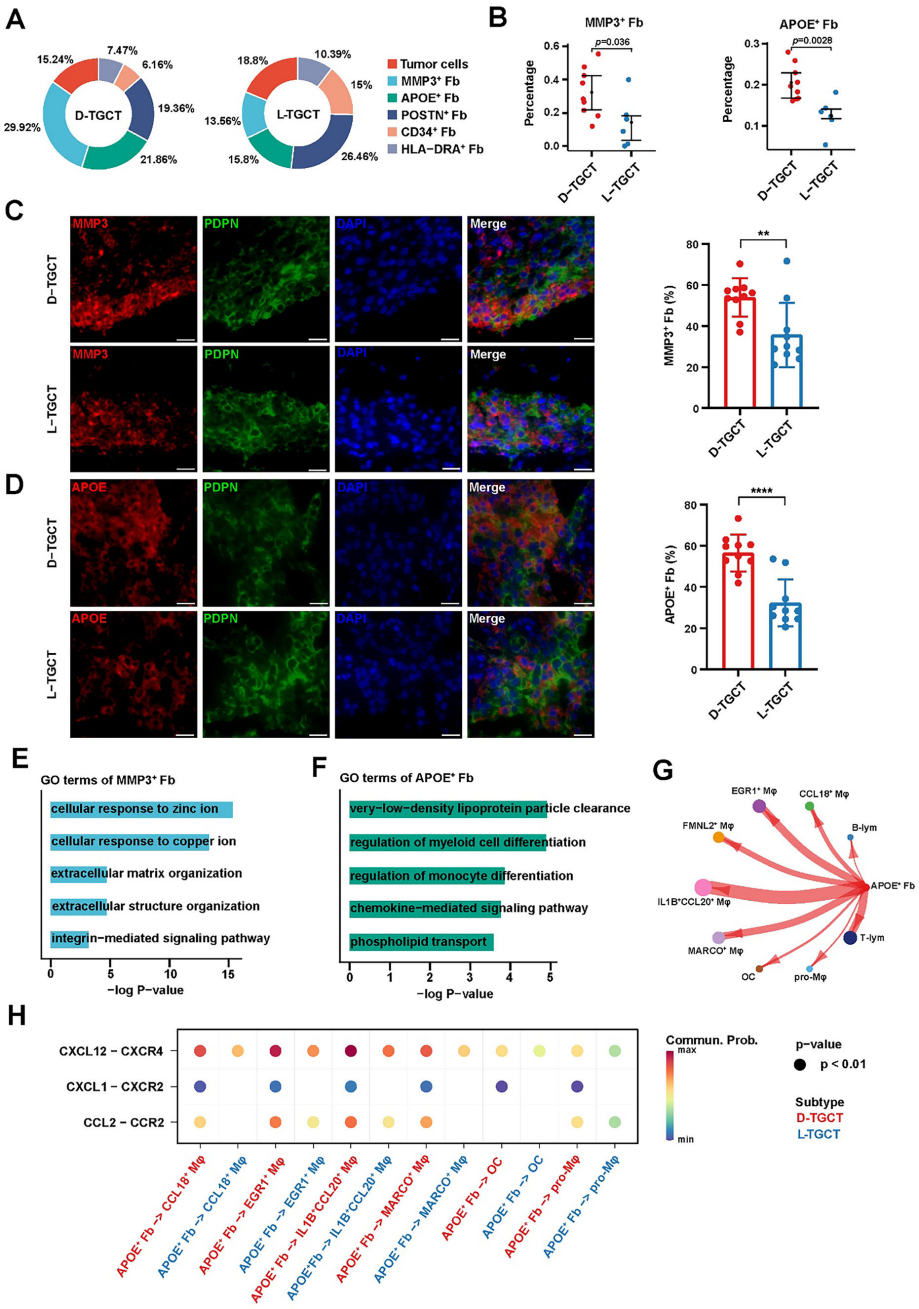
Higher proportions of *MMP3* ^+^ fibroblasts and *APOE* ^+^ fibroblasts were associated with locally aggressive behavior of D‐TGCT. A) Pie charts showing the percentages of different Fb subpopulations between the two types of TGCT. B) Box plots showing the differences in the proportions of *MMP3* ^+^ Fbs and *APOE* ^+^ Fbs between the two TGCT subtypes. Statistical significance was inferred by two‐sample *t* test. C) Representative mIHC staining of D‐TGCT (*n* = 10) and L‐TGCT (*n* = 10) lesions. Scale bar, 200 µm. Histogram on the right shows the percentages of *MMP3* ^+^ Fbs in D‐TGCT and L‐TGCT. D) Representative mIHC staining of D‐TGCT (*n* = 10) and L‐TGCT (*n* = 10) lesions. Scale bar, 200 µm. Histogram on the right shows the percentages of *APOE* ^+^ Fbs in D‐TGCT and L‐TGCT. E) GO enrichment analysis of *MMP3* ^+^ Fb marker genes. F) GO enrichment analysis of *APOE* ^+^ Fb marker genes. G) Circle plot showing the cell–cell communication between *APOE* ^+^ Fbs and other immune cells. Line thickness indicates the relative interaction intensity between D‐TGCT and L‐TGCT. H) Dot plot showing the ligand–receptor interaction of the chemokine pathway between *APOE* ^+^ Fb and Mφ subpopulations.

To gain further insight into the functional heterogeneity of Fbs, we re‐clustered them into six subpopulations (Figure S3A, Supporting Information) and annotated them by DEG expression level (Figure S3B, Supporting Information). Specifically, within these six subpopulations, we identified a subgroup expressing the molecular characteristics of TGCT tumor cells. Previous studies have shown that tumor cells in TGCT derive from Fbs.^[^ ^27^, ^28^ ^]^ A recent study has indicated the potential of *GFPT2* as a marker for tumor cells in TGCT and its association with activation of the Hippo signaling pathway.^[^ ^29^ ^]^ Consistent with those findings, we discovered that the above‐described Fb subpopulation indeed expressed higher levels of *GFPT2* (Figure S3D, Supporting Information) and scored higher on activation of the Hippo pathway (Figure S3E, Supporting Information) than other Fb subpopulations. In the above‐mentioned cell type annotations, we compared differences in proportions of the six identified Fb subpopulations between the two types of TGCT and discovered that the proportions of *MMP3* ^+^ Fbs and *APOE* ^+^ Fbs were significantly higher in D‐TGCT than in L‐TGCT (**Figure** **3**A,B), which we further validated via multiplex immunohistochemical (mIHC) staining (Figure 3C,D); these findings suggested that these Fbs played key roles in the development of D‐TGCT. *MMP3* is a secreted protein reported to be involved in cell invasiveness and cancer progression.^[^ ^30^ ^]^ GO functional enrichment analysis of *MMP3* ^+^ Fbs revealed functions of matrix remodeling and matrix degradation (Figure 5E), suggesting these Fbs’ potential role in local joint destruction. A previous study identified an Fb subpopulation in the synovial lining that was closely related to active rheumatoid arthritis (RA).^[^ ^31^ ^]^ Notably, *MMP3* ^+^ Fbs in our study also highly expressed marker genes associated with an Fb subpopulation closely related to active RA (Figure S3F, Supporting Information). Using these marker genes as a gene set of Fb activation, we found that *MMP3* ^+^ Fbs scored significantly higher than the other subpopulations (Figure S3G, Supporting Information), indicating their activated state and correlation with aggression of D‐TGCT.

For *APOE* ^+^ Fbs, enriched GO terms were related to functions of regulation in myeloid differentiation and chemokine‐mediated signaling pathway (Figure 3F). CCI analysis also suggested that *APOE* ^+^ Fbs had the strongest interactions with *IL‐1B* ^+^ *CCL20* ^+^ Mφs (Figure 3G), the major Mφ subpopulation related to the invasive ability of D‐TGCT reported in the previous subsection of this article. Specifically, of all ligand–receptor pairs in the chemokine and cytokine pathways, the CXCL12/CXCR4 axis had the highest communication probability between *APOE* ^+^ Fbs and *IL‐1B* ^+^ *CCL20* ^+^ Mφs (Figure 3H). Such communication has been reported to mediate the transformation of circulating monocytes into perivascular Mφs in the brain, causing neuroinflammation.^[^ ^32^ ^]^ Taken together, the above‐mentioned analyses suggested that *APOE* ^+^ Fbs in D‐TGCT might activate *IL‐1B* ^+^ *CCL20* ^+^ Mφs through the CXCL12/CXCR4 axis and that, together with *MMP3* ^+^ Fbs, they might contribute to the aggression of D‐TGCT.

### 2.4 Tumor Cells Promoted Differentiation of *CD34* ^+^ Fibroblasts into *MMP3* ^+^ Fibroblasts and *APOE* ^+^ Fibroblasts

**Figure 4:**
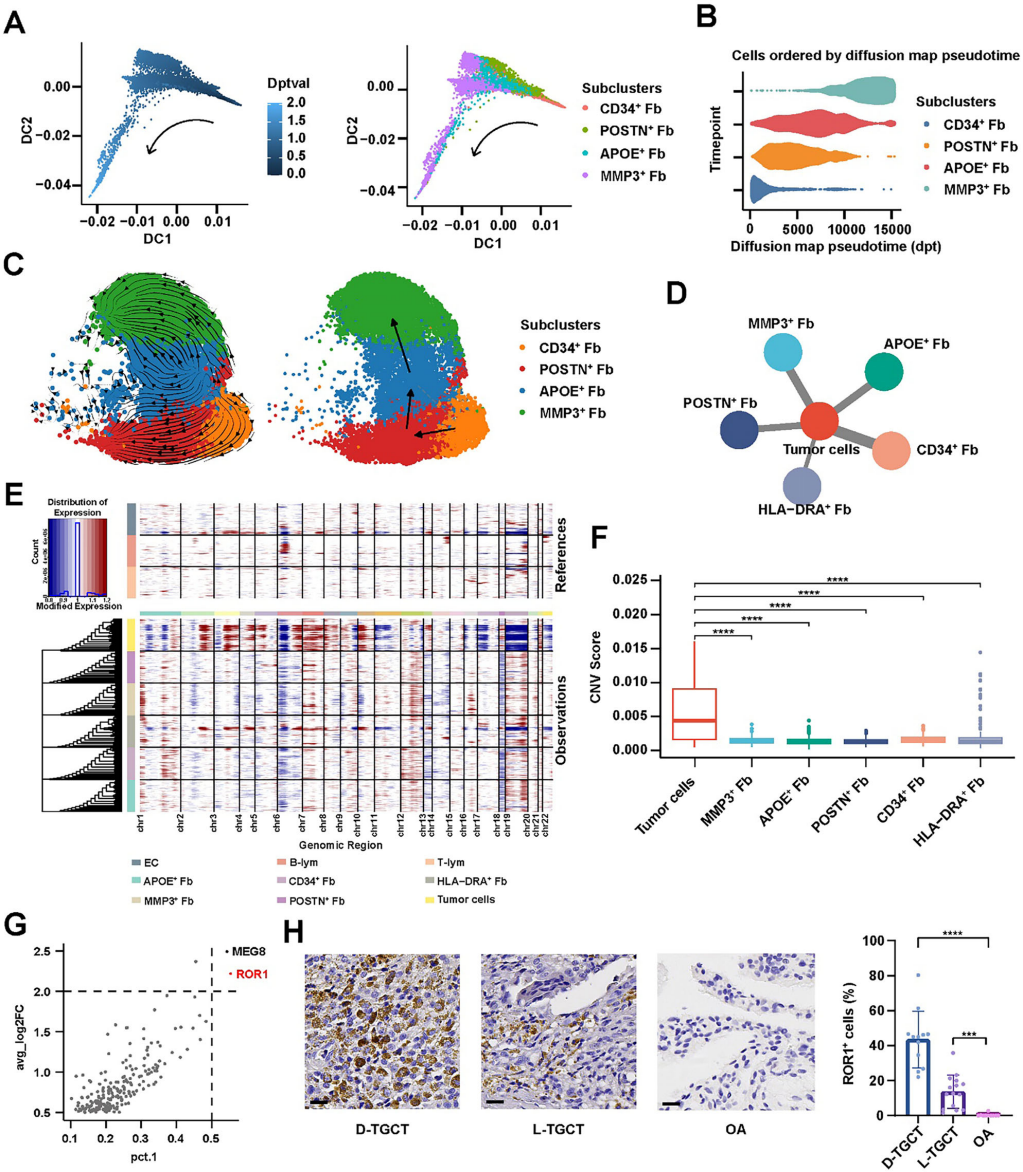
Identification of tumor cells and their specific markers. A) Diffusion maps visualizing the differentiation trajectories of *CD34* ^+^ Fbs, *POSTN* ^+^ Fbs, *APOE* ^+^ Fbs, and *MMP3* ^+^ Fbs. Cells were colored by inferred diffusion pseudotime (left) and subpopulation (right). B) *CD34* ^+^ Fbs, *POSTN* ^+^ Fbs, *APOE* ^+^ Fbs, and *MMP3* ^+^ Fbs ordered by diffusion map pseudotime. C) RNA velocities of *CD34* ^+^ Fbs, *POSTN* ^+^ Fbs, *APOE* ^+^ Fbs, and *MMP3* ^+^ Fbs visualized as a streamline plot (left) and a partition‐based graph abstraction (PAGA) plot (right) in a UMAP‐based embedding. D) Correlative network showing CCI based on scRNA‐seq and array data. Nodes represent different cell subpopulations. Line thickness indicates the strength of correlation. E) Heatmap showing the CNV profile of each Fb subpopulation. ECs, B cells, and T cells were selected as reference cells. Blue and red colors represent lost and amplified chromosomes, respectively. F) Box plot showing the CNV scores of different Fb subpopulations. G) Identification of *ROR1* as the marker gene for TGCT tumor cells. H) Representative IHC staining images of *ROR1* in D‐TGCT, L‐TGCT, and OA synovium. Scale bar, 20 µm. Histogram on the right shows the percentages of *ROR1* ^+^ cells in D‐TGCT, L‐TGCT, and OA synovium. Statistical significance was inferred by two‐sample *t* test. ****P < 0.0001, ***P < 0.001. Scale bar, 200 µm.

To further investigate why we observed higher proportions of *MMP3* ^+^ Fbs and *APOE* ^+^ Fbs in D‐TGCT, we drew a diffusion map of our scRNA‐seq cohort and reconstructed the differentiation trajectory of Fb subsets. The results showed that *CD34* ^+^ Fbs had the smallest diffusion mapping value (dptval) and were located at the beginning of the trajectory, whereas *APOE* ^+^ Fbs and *MMP3* ^+^ Fbs were developed at the end of the trajectory (**Figure** **4**A,B). In addition, RNA velocity showed a similar developmental trajectory, starting with *CD34* ^+^ Fbs and ending with *APOE* ^+^ Fbs and *MMP3* ^+^ Fbs (Figure 4C). We found a higher proportion of *CD34* ^+^ Fbs, which have been found to be fibroblast progenitors,^[^ ^33^ ^]^ in L‐TGCT than in D‐TGCT (Figure 3A; Figure S3C,D, Supporting Information). These results suggested a possible underlying mechanism in D‐TGCT regulating the differentiation of *CD34* ^+^ Fbs into *APOE* ^+^ Fbs and *MMP3* ^+^ Fbs.

Subsequently, we applied a computational model previously used in cancer research to further characterize this hypothetical regulatory mechanism.^[^ ^34^ ^]^ Using a public‐microarray dataset of TGCT,^[^ ^35^ ^]^ researchers can identify genes that exhibit strong correlations with high abundance of a particular cell population, such as *MMP3* ^+^ Fbs or *APOE* ^+^ Fbs. We speculated that these genes might potentially regulate changes in cell abundance of the specific cell population. When matching these highly correlated genes back to our scRNA‐seq data, we could thus infer cell types that highly expressed those genes and denote them as potential upstream regulators. We thereby established a correlative CCI network and identified strong interactions between tumor cells and Fbs that were *CD34* ^+^, *MMP3* ^+^, or *APOE* ^+^ (Figure 4D; and Table S4, Supporting Information). These analyses suggested that tumor cells might promote differentiation of *CD34* ^+^ Fbs into *APOE* ^+^ Fbs and *MMP3* ^+^ Fbs in D‐TGCT, turning “mild” fibroblast clusters into “aggressive” ones.

### 2.5 Tumor Cells in TGCT Exhibited Higher Copy Number Variation and were Characterized by Expression of *ROR1*

To detect the functional characteristics of tumor cells and reveal the specific mechanisms by which they regulated the development of aggressive Fbs, we first established a procedure to identify tumor cells and their specific markers. Chromosomal abnormalities, most commonly translocation of chromosome 1p11–13, have recently been characterized as the major pathogenic mechanisms in TGCT.^[^ ^17^ ^]^ Therefore, we suspected that quantifying chromosomal instability, such as copy number variation (CNV) events, in Fb subsets could effectively identify neoplastic cells. To that end, we initially performed CNV inference on all Fb subpopulations using inferCNV (Broad Institute, Cambridge, MA, USA), with ECs, T cells, and B cells selected as references (Figure 4E). Subsequently, the CNV score of each subset was calculated, and those with high scores were annotated as tumor cells (Figure 4F).

In a previous study, Van et al. identified tumor cells in TGCT using long‐read RNA‐seq (LR‐RNA‐seq) to detect the exact site of translocation.^[^ ^29^ ^]^ Based on the detected translocation event, they identified two Fb subsets as neoplastic TGCT cells. To assess the accuracy of our strategy of identifying tumor cells by inferring chromosomal CNVs, we reanalyzed Van et al.’s scRNA‐seq data and calculated CNV scores in two subsets of tumor cells identified via LR‐RNA‐seq. CNV scores in T cells and other Fbs were also calculated to provide control groups (Figure S4A,B, Supporting Information). Our analysis revealed that the CNV scores of the two tumor cell populations were indeed significantly higher than those of the control cell groups (Figure S4C, Supporting Information), indicating that identifying tumor cells in TGCT using inferCNV was accurate and feasible.

Next, we attempted to identify the marker gene of TGCT tumor cells based on the following criteria: (1) expressed in <3% of non‐tumor cells (pct.2 < 0.03); (2) expressed in >50% of tumor cells (pct.1 > 0.50); and (3) |log2 fold change (FC)| > 2 when comparing expression levels between tumor and non‐tumor cells. Ultimately, two genes, maternally expressed gene 8 (*MEG8*) and receptor tyrosine kinase–like orphan receptor 1 (*ROR1*), met these criteria (Figure 4G). We selected *ROR1* as the potential marker gene for tumor cells because *MEG8* is a long non‐coding RNA (lncRNA), whereas *ROR1* encodes a cell surface receptor closely related to tumor growth, invasion, and metastasis.^[^ ^36^, ^37^, ^38^ ^]^ In addition, we compared the expression levels of *ROR1* in other synovial samples and found that ROR1 was rarely expressed in Fbs from OA synovium (Figure S4D, Supporting Information). To verify the above findings, we performed immunohistochemical (IHC) staining of *ROR1* in the discovery cohort (Figure S4E, Supporting Information) and in an external cohort consisting of 14 D‐TGCT, 12 L‐TGCT, and 10 OA‐synovium samples (Figure 4H). A significantly higher proportion of *ROR1* ^+^ cells was observed in both D‐TGCT and L‐TGCT than in OA synovium, demonstrating that *ROR1* was a specific marker of tumor cells for TGCT.

### 2.6 Invasion‐Related Subpopulations of Tumor Cells (meta‐program 3) were Specifically Enriched in D‐TGCT

**Figure 5:**
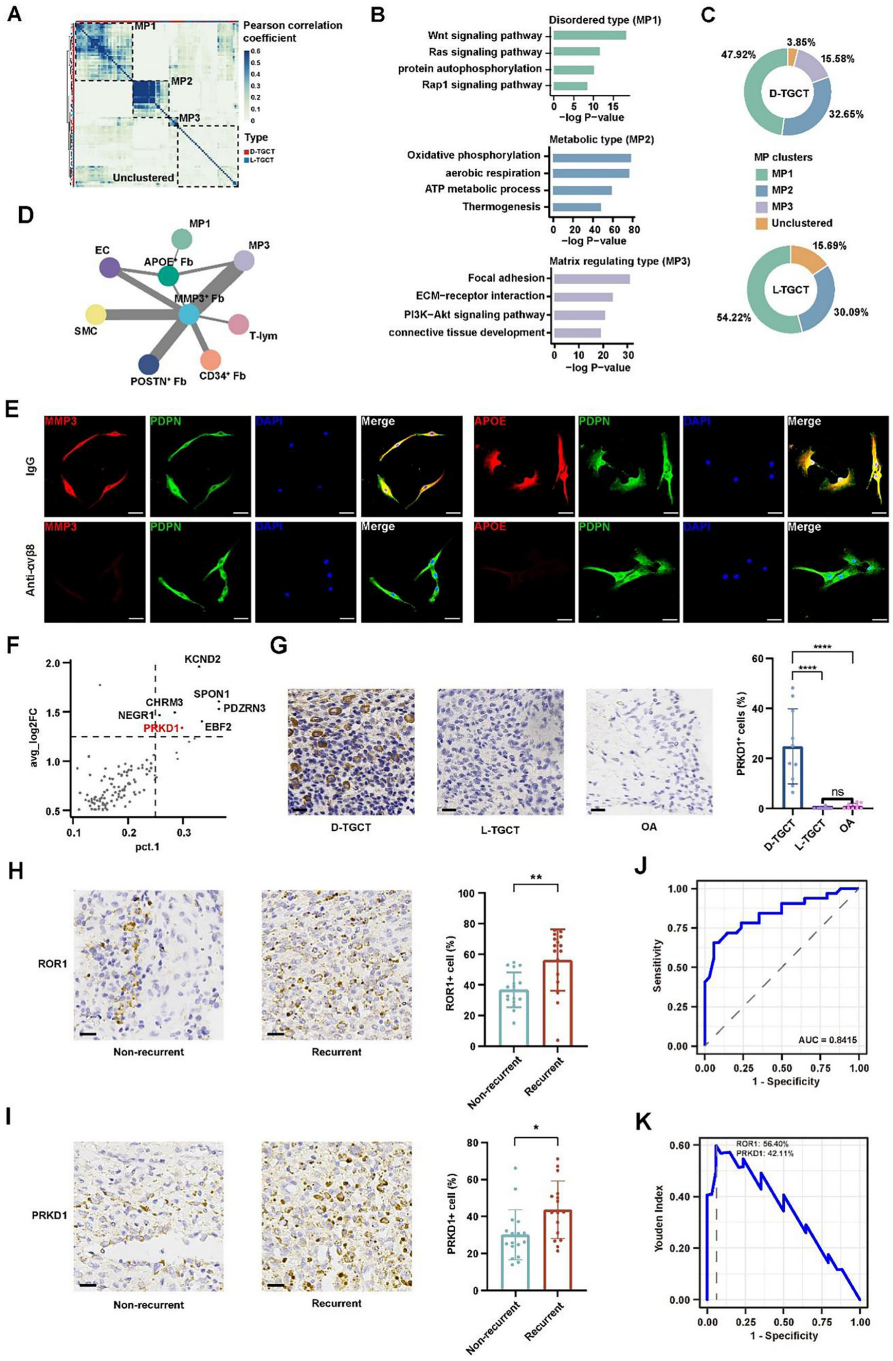
Identification of invasion‐related subpopulations of tumor cells (meta‐program 3) and the ability of *ROR1* and *PRKD1* to predict recurrence. A) Heatmap showing PCC among 94 sample‐level programs, calculated based on their top 50 marker genes. Programs were clustered into three MPs with similar functions (framed by black dashed lines). B) GO and KEGG enrichment analysis of the three identified MPs. C) Pie charts showing the proportions of MPs between D‐TGCT and L‐TGCT. D) Correlative network showing CCI based on scRNA‐seq and array data. Nodes represent different tumor cell MPs or other cell subpopulations. Line thickness indicates the strength of correlation. E) Representative mIHC images of *CD34* ^+^ Fbs treated with CM of synoviocytes from D‐TGCT in the absence (top) and presence (bottom) of integrin αvβ8 receptor Ab. Scale bar, 50 µm. F) Identification of *PRKD1* as the marker gene for MP3 tumor cells. G) Representative IHC staining images of *PRKD1* in D‐TGCT, L‐TGCT, and OA synovium. Scale bar, 20 µm. Histogram on the right shows the percentages of *PRKD1* ^+^ cells in D‐TGCT, L‐TGCT, and OA synovium. Statistical significance was inferred by two‐sample *t* test. ****P < 0.0001, ns = not significant. H) Representative IHC staining images of *ROR1* in non‐recurrent (*n* = 17) and recurrent (*n* = 16) D‐TGCT. Scale bar, 20 µm. Histogram on the right shows the percentages of *ROR1* ^+^ cells in non‐recurrent and recurrent D‐TGCT. Statistical significance was inferred by two‐sample *t* test. ***P < 0.001. I) Representative IHC staining images of *PRKD1* in non‐recurrent (*n* = 17) and recurrent (*n* = 16) D‐TGCT. Scale bar, 20 µm. Histogram on the right shows the percentages of *PRKD1* ^+^ cells in non‐recurrent and recurrent D‐TGCT. Statistical significance was inferred by two‐sample *t* test. *P < 0.05. J) Receiver operating characteristic (ROC) curve evaluating the performance of *ROR1* and *PRKD1* in predicting disease recurrence. K) Determination of the threshold for *ROR1* and *PRKD1* positivity rates using the Youden index.

Next, we explored the functional characteristics of tumor cells by calculating their signature scores in TGCT samples. Surprisingly, we found that TGCT tumor cells had lower malignant‐tumor scores, such as on necroptosis, pro‐metastatic properties, and proliferation, than the other Fb subsets (Figure S5A, Supporting Information), as well as on orthopedic malignant tumor, such as osteosarcoma^[^ ^39^ ^]^ (OS; Figure S5B, Supporting Information) and Ewing sarcoma^[^ ^40^ ^]^ (ES; Figure S5C, Supporting Information). To further characterize the functional characteristics of TGCT, we conducted consensus non‐negative matrix factorization (cNMF) analysis of tumor cells from primary TGCT. This unsupervised clustering approach generated 94 programs, each composed of tumor cells with similar functions (**Figure** **5**A). Subsequently, all programs were clustered into three meta‐programs (MPs) based on the Pearson's correlation coefficient (PCC) of their top 50 genes (Figure 5A). To investigate the biological function of each meta‐program, we performed functional enrichment analysis of the DEGs in each MP. Specifically, the GO and Kyoto Encyclopedia of Genes and Genomes (KEGG) terms enriched for MP1 were Wingless/Integrated (Wnt) signaling pathway, *Ras* signaling pathway, protein autophosphorylation, and Ras‐related protein 1 (*Rap1*) signaling pathway; those for MP2 were oxidative phosphorylation (OXPHOS), aerobic respiration, adenosine triphosphate (ATP) metabolic process, and thermogenesis; and those for MP3 were focal adhesion, extracellular‐matrix (ECM)–receptor interaction, phosphoinositide 3‐kinase (PI3K)/protein kinase B (Akt) signaling pathway, and connective‐tissue development (Figure 5B). Accordingly, we annotated these three MPs as disordered type (MP1), metabolic type (MP2), and matrix regulating type (MP3), respectively. Notably, MP3 were exclusive to D‐TGCT (Figure 5C), implying their pivotal role in influencing the phenotype of this disease subtype.

Similarly, we conducted correlative network analysis between the three MPs and other cell types and identified the strongest interactions between MP3 tumor cells and *MMP3* ^+^ Fbs (Figure 5D; and Table S5, Supporting Information). Interestingly, when we placed *APOE* ^+^ Fbs at the center of the network, we also observed strong interaction between MP3 tumor cells and *APOE* ^+^ Fbs (Figure 5D; and Table S5, Supporting Information). Furthermore, CCI analysis performed using CellChat identified the COL6A3 − (ITGAV + ITGB8) interaction, namely COL6A3 − integrin αvβ8, to have the highest communication probability, which might be the mechanism regulating the formation of *APOE* ^+^ Fbs and *MMP3* ^+^ Fbs (Figure S5D,E, Supporting Information). To further verify the above analytic results, we performed a fibroblastic‐differentiation assay. After being treated with the conditioned medium (CM) of D‐TGCT synoviocytes, *CD34* ^+^ Fbs expressed higher levels of *MMP3* and *APOE* than those treated with CM and integrin αvβ8 receptor antibody (Ab; Figure 5E), while *CD34* ^+^ Fbs treated with COL6A3 recombinant protein (r‐COL6A3) expressed higher levels of *MMP3* and *APOE* than those treated with CM only (Figure S5F, Supporting Information). This suggested that COL6A3 promoted differentiation of *CD34* ^+^ Fbs toward *APOE* ^+^ Fbs and *MMP3* ^+^ Fbs and that integrin αvβ8 receptor Ab inhibited this differentiation.

Using the same criteria mentioned above, we attempted to identify markers for MP3 tumor cells. A total of seven genes were obtained (Figure 5F); of these, PRKD1, also known as serine/threonine‐protein kinase D1, is reported to play a crucial role in promoting acinar‐to‐ductal metaplasia,^[^ ^41^ ^]^ wound healing, and skin tumor formation^[^ ^42^ ^]^ closely related to cancer progression. Therefore, we hypothesized that PRKD1 might also have a tumor‐promoting function in D‐TGCT and could potentially serve as a marker gene for MP3 tumor cells. Moreover, the low expression of PRKD1 in OA synovium further supported this gene's diagnostic significance (Figure S5G, Supporting Information). To validate the aforementioned findings, we performed IHC staining of PRKD1 in the discovery cohort (Figure S5H, Supporting Information) and in an external cohort consisting of 10 D‐TGCT, 10 L‐TGCT, and 10 OA‐synovium samples (Figure 5G). Concordantly, the results revealed a significantly higher percentage of *PRKD1* ^+^ cells in D‐TGCT than in L‐TGCT and OA synovium, thereby confirming *PRKD1* as a reliable marker for MP3 tumor cells.

In short, we identified an exclusive tumor cell subpopulation MP3 in D‐TGCT and confirmed the role of these tumor cells in regulating differentiation of *CD34* ^+^ Fbs toward *APOE* ^+^ Fbs and *MMP3* ^+^ Fbs through COL6A3 − (ITGAV + ITGB8) interaction. Targeting integrin αvβ8 receptor could be a potential treatment strategy to prevent destruction of bone and cartilage in D‐TGCT.

### 2.7 The Potential Roles of *ROR1* and *PRKD1* in Predicting D‐TGCT Recurrence

Interestingly, when comparing recurrent samples with primary samples, we observed significantly higher expression levels of *ROR1* and *PRKD1* in the former, suggesting the potential role of *ROR1* and *PRKD1* in predicting D‐TGCT recurrence (Figure S5H,I, Supporting Information). To validate this hypothesis, we conducted a follow‐up study on D‐TGCT patients who had undergone surgical resection. Postoperative pathological sections of primary lesions were collected for IHC staining from patients experiencing disease recurrence, while those without recurrence served as the control group. Sixteen D‐TGCT patients who developed relapse eventually underwent follow‐up. IHC staining revealed markedly elevated expression levels of *ROR1* and *PRKD1* in the primary lesions of these patients compared with those of non‐recurrent patients (Figure 5H,I), indicating the high predictive potential of these two markers for D‐TGCT recurrence. The area under the curve (AUC) for predicting D‐TGCT recurrence using *ROR1* and *PRKD1* was 0.8415 (Figure 5J). We then calculated the Youden index and determined the maximum value as the optimal cutoff. Finally, we identified that a primary lesion of a TGCT patient with a *ROR1* ^+^ rate >56.40% and a *PRKD1* ^+^ rate >42.11% was at risk of recurrence (Figure 5K).

## 3 Discussion

**Figure 6:**
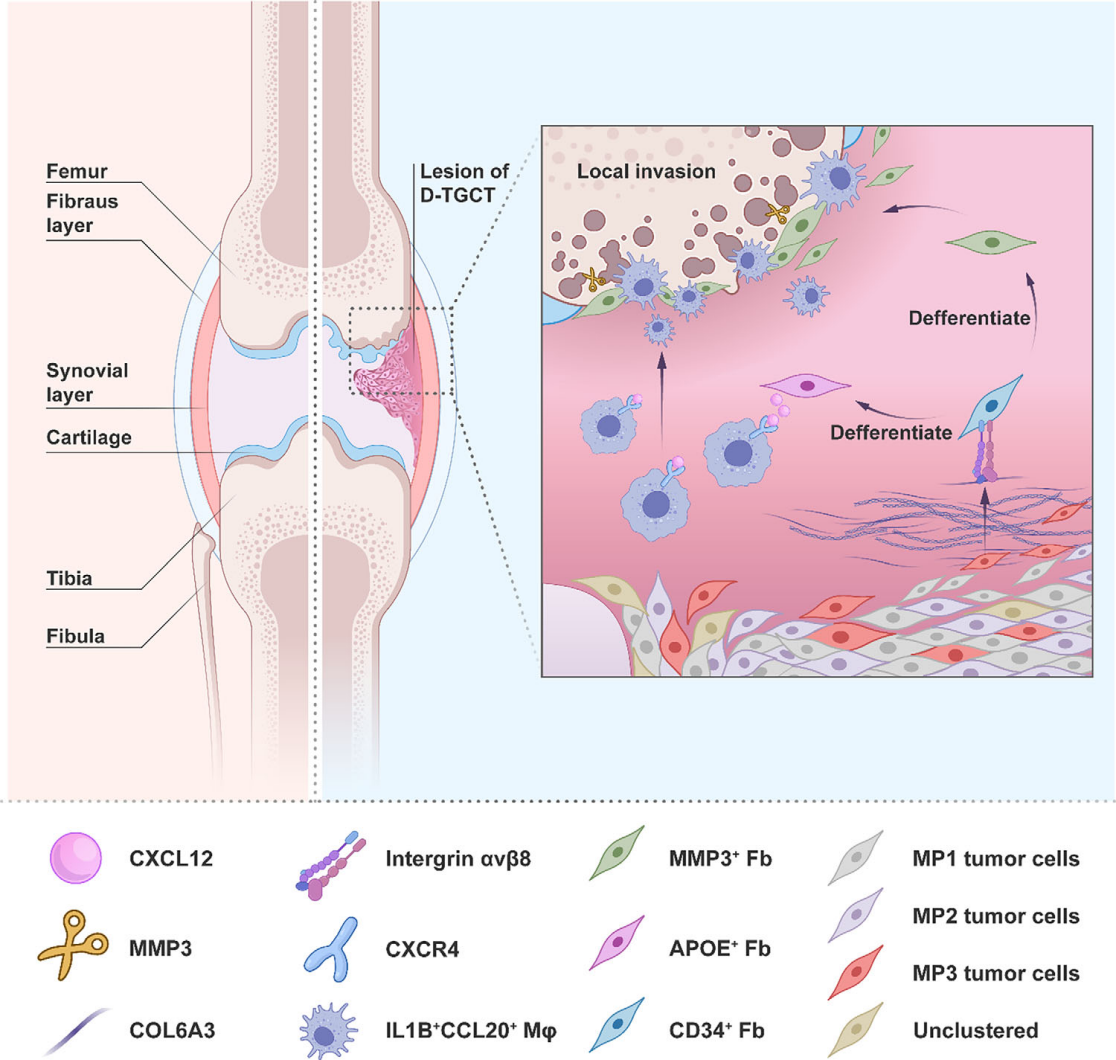
Schematic diagram of local invasion in D‐TGCT. MP3 tumor cells, a specific subpopulation of tumor cells in D‐TGCT, regulated the differentiation of *CD34* ^+^ Fbs into *MMP3* ^+^ Fbs and *APOE* ^+^ Fbs through COL6A3 − (ITGAV + ITGB8) interaction. *APOE* ^+^ Fbs activated *IL‐1B* ^+^ *CCL20* ^+^ Mφs through the CXCL12/CXCR4 axis. *IL‐1B* ^+^ *CCL20* ^+^ Mφs and *MMP3* ^+^ Fbs participated in the local invasion of D‐TGCT.

In the current study, we conducted scRNA‐seq analysis to explore TME divergence between D‐TGCT and L‐TGCT. In our integrated analysis, we discovered a unique subpopulation of tumor cells (MP3 cluster) in D‐TGCT that could regulate differentiation of *CD34* ^+^ Fbs into *MMP3* ^+^ Fbs and *APOE* ^+^ Fbs through the COL6A3 − (ITGAV + ITGB8) ligand–receptor pair. We also found that *APOE* ^+^ Fbs could activate *IL‐1B* ^+^ *CCL20* ^+^ Mφs through the CXCL12/CXCR4 ligand–receptor pair. *IL‐1B* ^+^ *CCL20* ^+^ Mφs, along with *MMP3* ^+^ Fbs, participated in the aggressive behavior of D‐TGCT (**Figure** **6**). We also identified and validated two effective biomarkers, *ROR1* and *PRKD1*, to predict disease recurrence.

Compared with L‐TGCT, D‐TGCT significantly impairs patients’ QoL due to its aggressive nature and high recurrence rate.^[^ ^43^, ^44^ ^]^ Our previous findings suggested that the inflammatory factors IL‐1β and tumor necrosis factor‐α (TNF‐α) were enriched in D‐TGCT synovial fluid, which could upregulate the expression of cadherin‐11 through the PI3K/Akt signaling pathway. Elevated expression of cadherin‐11 can further promote the growth, migration, and invasion of Fb‐like synoviocytes in D‐TCGT.^[^ ^45^ ^]^ Consistently, in our scRNA‐seq data, pathway analysis of MP3 tumor cells with core regulatory functions in D‐TGCT revealed activation of PI3K/Akt signaling (Figure 5B). We also found that the MP3 subpopulation highly expressed the *CDH11* transcript, which encodes cadherin‐11 (Figure S3H, Supporting Information). This result further supported the functional importance of the MP3 subpopulation in D‐TGCT. Therefore, targeting integrin αvβ8 in the MP3 subpopulation might hold promise for preventing progression of D‐TGCT and destruction of the surrounding tissues. Recently, several selective inhibitors have been developed for integrin αvβ8. For example, Reichart et al. developed a cyclic octapeptide c(GLRGDLpP) that selectively targets integrin αvβ8.^[^ ^46^ ^]^ Roy et al.^[^ ^47^ ^]^ employed computational methods to design inhibitors with optimal binding properties to specifically target integrins αvβ6 and αvβ8, the efficacy of which was validated by binding assays. In the future, further research into the therapeutic application of these compounds in D‐TGCT will be highly worthwhile.

Based on the percentage expression of cells in a given cluster, the log FC value, and the corresponding biological function, we ultimately identified *ROR1* as a marker for tumor cells in TGCT and *PRKD1* as a marker for MP3 tumor cells in D‐TGCT. *ROR1* is reported to be detectable in embryonic tissue only and is generally absent in adult tissue.^[^ ^48^ ^]^ As reported in recent studies, *ROR1* is selectively overexpressed in several solid and hematological malignancies, including ovarian cancer, triple‐negative breast cancer, and lung adenocarcinomas, making it a promising therapeutic target for cancer treatment.^[^ ^49^, ^50^, ^51^ ^]^ In addition, activated *PRKD1* is involved in regulating a variety of cellular functions, especially those related to malignant transformation, such as cell proliferation, growth, migration or invasion, apoptosis, and epidermal–mesenchymal transition.^[^ ^52^, ^53^, ^54^ ^]^ This study was the first to uncover the specific expression of *PRKD1* in MP3 tumor cells, prompting the reasonable speculation that *PRKD1* plays a significant role in the pathological progression of D‐TGCT. Stewart et al.^[^ ^55^ ^]^ found that resveratrol inhibits autophosphorylation of *PRKD1* in a concentration‐dependent manner, thereby inhibiting its activation. Whether treatment with resveratrol can delay local invasion and recurrence by inhibiting proliferation of MP3‐type tumor cells in D‐TGCT might be worth further exploring.

This study has several limitations. First, the number of recurrent TGCT samples used for scRNA‐seq was insufficient. Although we used IHC staining to demonstrate the ability of *ROR1* and *PRKD1* to predict TGCT recurrence, transcriptomic differences between recurrent and primary TGCT were not fully explored. The potential mechanisms mediating the recurrence of TGCT and the roles of *ROR1* and *PRKD1* in the process of recurrence remain unclear, impeding the identification of effective preventive targets for recurrence. Second, there are current shortages of TGCT tumor cell lines and animal models in the field, which hamper the in‐depth investigation of its molecular mechanisms and restricts the validation of potential therapeutic targets. Establishment of tumor cell lines and animal models is therefore imperative for further research.

In conclusion, this study created a comprehensive single‐cell transcriptomic atlas of D‐TGCT and L‐TGCT, identifying novel cell subpopulations and cellular interactions related to the aggressive behavior of D‐TGCT. Our findings deepened the understanding of the cellular composition and pathogenic mechanisms of TGCT, shedding light on the disease's diagnosis, treatment, and prognosis.

## 4 Experimental Section

### Patient Samples

For scRNA‐seq, surgically resected specimens were collected from 17 TGCT patients diagnosed by preoperative needle biopsy and MRI, including 9 primary D‐TGCT, 1 recurrent D‐TGCT, 6 primary L‐TGCT, and 1 recurrent L‐TGCT, as a discovery cohort. All TGCT patients had no history of radiotherapy or systemic therapy. 3 synovium tissues from OA patients were collected as one of the validation cohorts. Paraffin‐embedded sections from 12 D‐TGCT, 15 L‐TGCT, and 10 OA synovium were acquired for IHC staining. Paraffin‐embedded sections from 10 D‐TGCT and 10 L‐TGCT were acquired for mIHC staining.

TGCT patients who received surgical resection were followed up for at least two years. For patients who underwent recurrence, paraffin‐embedded sections of their paired primary lesion were collected, and those of recurrence‐free patients were enrolled as controls. A total of 20 sections from recurrent TGCT (16 D‐TGCT, 4 L‐TGCT) and 20 controls (17 D‐TGCT, 3 L‐TGCT) were obtained for IHC staining of recurrence‐associated markers.

This study was approved by the Ethics Committee of Sun Yat‐Sen Memorial Hospital, Sun Yat‐Sen University (SYSKY‐2023‐906‐01). Informed consents were obtained from all patients participating in this study.

### Single‐Cell Suspension Preparation

Fresh TGCT lesions were sliced into 1 mm^3^ pieces, washed with phosphate‐buffered saline (PBS, Gibco) 2 times, and then digested with Collagenase I (Diamond, cat# A004194‐0001) and Hyaluronidase (Solarbio, cat# H8030) on a shaker (100 r.p.m) at 37 °C for 30 min. The cell suspension was filtered and collected through a 70 µm cell strainer. The rest of the tissues were digested with trypsin (Gibco, cat# 25 200 056) in the aforementioned condition.

### scRNA‐seq and Raw Data Processing

For TGCT, prepared single‐cell suspensions were loaded to generate single‐cell gel bead‐in‐emulsions (GEMs) using Chromium Next GEM Single Cell 5′ Reagent Kits v2 (10x Genomics). The 5′ gene expression and the V(D)J libraries were constructed according to the manufacturer's protocol and then sequenced on NovaSeq 6000 (Illumina). Raw sequencing data were mapped to the GRCh38 human reference genome using CellRanger (version 7.0.1) to generate a gene expression matrix of each sample. Next, the R package SoupX (version 1.6.2)^[^ ^56^ ^]^ was utilized to estimate and remove ambient RNA contamination.

BD Rhapsody system was used to capture the transcriptome of single cells from OA synovium. The raw data were aligned to the GRCh38 human reference genome using the Common Workflow Language (cwl) pipelines to generate a gene expression matrix of each sample for further analysis.

Cells with more than 200 features, less than 6000 features, and less than 10% mitochondrial genes were retained as high‐quality cells. Cells that coexpressed marker genes of two or more distinct cell lineages were regarded as doublets and removed (eg, cells coexpressed T cell marker CD3D and B cell marker CD19). Finally, a total of 123970 cells from TGCT and 10797 cells from OA synovium were preserved.

### Dimensional Reduction and Unsupervised Clustering

R package Seurat (version 4.3.0)^[^ ^57^ ^]^ was applied for standard scRNA‐seq data analysis. Briefly, normalization of the gene‐cell count matrix, scaling, and identification of highly variable genes were conducted with default parameters. The optimal number of principal components (PCs) was determined by the ElbowPlot function. R package harmony (version 0.1.1)^[^ ^58^ ^]^ was applied to remove the batch effect. The top 3200 variable genes and the first 15 PCs were used for unsupervised clustering analysis with a resolution set to 1.5. The uniform manifold approximation and projection (UMAP) were further conducted for cluster visualization. The second round of subclustering was performed with suitably adjusted parameters.

### Differential Expression, Pathway Analysis, and Calculation of Signature Scores

The “FindAllMarkers” and “FindMarkers” functions from Seurat were utilized to identify differentially expressed genes (DEGs) with the parameter “min.pct” set to 0.1, “logfc.threshold” set to 0.5, and “only.pos” set to TRUE. DEGs with adjusted p‐values < 0.05 were used for enrichment analysis. Gene Ontology (GO) and Kyoto Encyclopedia of Genes and Genomes (KEGG) pathway enrichment analysis of the DEGs were performed using the R package clusterProfiler (version 4.8.1).^[^ ^59^ ^]^ The enriched pathway with a *p*‐value <0.05 was considered significant. The signature scores of each cell were calculated using the “AddModuleScore” function in Seurat.

### Inferring CNV from scRNA‐seq

Since tumor cells of TGCT are characterized by the genomic aberrations involving CSF1, the chromosomal copy number variation (CNV) of the tumor cells is higher than other cells. The endothelial cells, T lymphocytes, and B lymphocytes were used as references, and compared the gene expression of each fibroblast subpopulation with that of the references to infer the chromosomal CNV using the R package infercnv (version 1.14.2).^[^ ^60^, ^61^ ^]^ The gene expression of each fibroblast was then normalized to a scale of ‐1 to 1, and the sum of squares of the normalized values was defined as the CNV score. Cells with higher CNV scores were annotated as tumor cells.

### Defining Robust cNMF Programs and MPs

The principle of the consensus non‐negative matrix factorization (cNMF) was to factorize the cells × genes matrix into cells × programs matrix and programs × genes matrix. The correlation of gene expression was calculated to cluster programs so as to classify cells into several functionally distinct meta‐programs (MPs). The “cnmf factorize” function from the cNMF analysis pipeline (version 1.4.1)^[^ ^62^ ^]^ was used to factorize the expression matrix of tumor cells from primary TGCT. All samples were sequentially decomposed into 3 to 10 components (K), with 300 iterations each time, and all results were merged using the “cnmf combine” function. The suitable K value of each sample was determined based on the stability and error. Finally, each sample was divided into K programs, and each program contained several tumor cells with similar functions. All programs were clustered into three MPs according to the Pearson correlation coefficient.

After clustering the MPs, DEGs were calculated using the “FindAllMarkers” function with the parameter “min.pct” set to 0.1, “logfc.threshold” set to 1, and “only.pos” set to TRUE. Both GO and KEGG enrichment analyses of the DEGs were performed, and representative pathways with a p‐value <0.05 were selected for visualization to functionally annotate MPs.

### Construction of Pseudotime Trajectory

The seurat objects of *MMP3* ^+^ Fb, *APOE* ^+^ Fb, *CD34* ^+^ Fb, and *POSTN* ^+^ Fb were used to construct the pseudotime trajectory. The dimensionality reduction was generated using the “reducedDim” function from R package SingleCellExperiment (version 1.22.0),^[^ ^63^ ^]^ which was subsequently utilized for creating the diffusion map value using the “DiffusionMap” from R package destiny (version 3.14.0).^[^ ^64^ ^]^ The pseudotime trajectory was constructed by the “DPT” function and was visualized by the “ggplot” function from R package ggplot2 (version 3.4.2). The codes and parameters applied for macrophages subsets were identical.

### RNA Velocity

The expression matrix, gene names, and dimensionality reduction of *MMP3* ^+^ Fb, *APOE* ^+^ Fb, *CD34* ^+^ Fb, and *POSTN* ^+^ Fb were utilized to generate AnnData. The scRNA‐seq bam files of each sample were converted to loom files using python package velocyto (version 0.17.17),^[^ ^65^ ^]^ which were subsequently merged with the AnnData. The RNA velocities were calculated using the “scv.pl.velocity” function from R package scVelo (version 0.3.1)^[^ ^66^ ^]^ with parameter “mode” set to stochastic and were visualized using the “scv.tl.velocity_graph” function, “scv.pl.velocity_embedding_stream” function, and “scv.pl.paga” function. The codes and parameters applied for macrophages subsets were identical.

### Construction of Correlative Cell‐Cell Interactions and Cellular Interaction Analysis

First, the specific gene signatures for each interested cell type were calculated using the “FindMarkers” function (with parameter “logfc.threshold” set to 0.5, “min.pct” set to 0.1, “only.pos” set to TRUE) and the top 30 signatures with the maximum “avg_log2FC” were kept. Second, the “self‐expressed genes” of each cell type were identified based on the following criteria: 1) average expression > 1; 2) cell frequency of expression > 20%. Third, a TGCT microarray from the Gene Expression Omnibus was used to evaluate the abundance of cell types by the average expression of z‐score normalized log‐transformed expression of the cell type‐specific genes defined above. The Pearson correlation coefficient between the expression of each gene and the relative abundance of each cell type were computed with the correlation of “self‐expressed genes” transformed to 0 to obtain an adjusted correlation matrix. The top 30 genes with the largest Pearson correlation coefficient were selected as a gene set to calculate the enrichment score using the “AddModuleScore” function. The correlative cell‐cell interactions were constructed according to the enrichment score using Cytoscape.

Potential cell‐cell interactions between different cell types were evaluated using R package CellChat (version 1.6.11).^[^ ^67^ ^]^ All CellChatDB databases were utilized to infer cellular communications. Briefly, the normalized data matrix of interested cell types was used to create CellChat object. Next, standardized processing functions such as “identifyOverExpressedGenes”, “identifyOverExpressedInteractions”, and “computeCommunProb”, etc were applied to identify interaction pairs. Communications involving less than 10 cells were filtered. Finally, the interaction strength and significant ligand‐receptor pairs from selected cell groups, such as MP3 and *MMP3* ^+^ Fb, MP3, and *APOE* ^+^ Fb, were visualized using the “netVisual_circle” function and the “netVisual_bubble” function.

### Multiplex Immunohistochemistry Staining

Formalin‐fixed, paraffin‐embedded blocks of TGCT tissue were sectioned into 4 µm slides, deparaffinized in xylene, rehydrated with 100%, 95%, and 75% ethanol, and fixed in 10% neutral buffered formalin. Boiled 1x EDTA buffer (pH9.0) was used to retrieve the antigen. After blocking with 10% goat serum at room temperature for 1 h, anti‐Podoplanin antibody (Abcam, Cat# Ab236529), anti‐MMP3 antibody (Abcam, Cat# Ab53015), anti‐Apolipoprotein E antibody (Abcam, Cat# Ab183597), and anti‐CD34 antibody (Abcam, Cat# Ab315802) were used sequentially at 4 °C for ≈12 h. Sections were then incubated with HRP‐conjugated secondary antibody at room temperature for 30 min. Tyramide signal amplification was performed using fluorescent dye TG650 (TissueGnostics, corresponding to anti‐Podoplanin antibody), TG570 (TissueGnostics, corresponding to anti‐MMP3 antibody), TG520 (TissueGnostics, corresponding to anti‐Apolipoprotein E antibody), and TG540 (TissueGnostics, corresponding to anti‐CD34 antibody). Nuclei were stained with DAPI (Panovue, Cat# 00 121 00500) for 10 min at room temperature. Stained sections were scanned using the TissueFaxs platform (TissueGnostics), and images were processed using the Strataquest software (Tissueg Nostics, V.7.1.1.119).

### Immunohistochemistry Staining

For ROR1 and PRKD1 IHC analysis, 4 µm formalin‐fixed, paraffin‐embedded sections were used. After deparaffinization and hydration, the sections were heated in antigen retrieval solution (1x EDTA buffer, pH9.0) and then incubated with 3% hydrogen peroxide to block endogenous peroxidases. Next, the sections were blocked with 10% goat serum (Solarbio, Cat# SL038‐10) for 30 min and subsequently incubated with anti‐ROR1 antibody (Abcam, Cat# Ab111174) or anti‐PRKD1 antibody (Abcam, Cat# Ab172096; Proteintech China, Cat# 20714‐1‐AP) at 4 °C overnight. Finally, the sections were probed with the secondary antibody and scanned with a KFPRO slicing scanner, and the pictures were analyzed and processed with Qupath (Version 0.3.1).

### Flow Cytometry Cell Sorting and Fibroblast Differentiation Assay

Single‐cell suspension was prepared as mentioned above. For surface staining, antibodies diluted in 2% fetal bovine serum (FBS) in PBS staining buffer were added to the suspension and incubated at room temperature for 20 min. Antibodies and the corresponding fluorochromes used were: CD45‐FITC (Biolegend, Cat# 304 005), CD31‐BU785 (Biolegend, Cat# 303 147), PDPN‐PE (Biolegend, Cat# 337 003), and CD34‐ PE/Cyanine7 (Biolegend, Cat# 343 515). The Flow cytometry was performed on AURORA/NL with SpectroFlo (CYTEK).

After sorting CD45^−^CD31^−^PDPN^+^CD34^+^ cells, 1 ml cell suspension was seeded on coverslip in a 24‐well plate for 24 h. Then the seeded cells were incubated with cell culture supernatant from D‐TGCT, 0.6 µg mL^−1^ integrin αvβ8 receptor antibody (Sigma‐Aldrich, Cat# ZRB1192), or 100 ng mL^−1^ COL6A3 recombinant protein (Abcam, Cat #ab276722) for 24 h before immunofluorescence staining.

### Immunofluorescence Staining

Cells seeded on coverslip in the 24‐well plate were fixed with 4% paraformaldehyde at 4 °C for 20 min and then incubated with 3% hydrogen peroxide. For each staining cycle, cells were blocked with 10% goat serum for 15 min and subsequently incubated with the corresponding primary antibody at 37 °C for 30 min. After incubation with the second antibody and fluorescent dye, nuclei were stained with DAPI for 5 min at room temperature. Finally, the fluorescence was quenched and the stained coverslip was heated in a 37 °C heater for 1 h. Antibodies and fluorescent dyes were identical to those employed in mIHC staining

### Construction of ROC Curve

To evaluate the efficacy of ROR1 and PRKD1 in predicting recurrence, a combination of the positive rate of these two markers was first established and then calculated the sensitivity and specificity under each combination. The best predictive cut‐off value was determined by calculating the Youden Index.

### Statistical Analysis

Statistical analyses were performed using R (version 4.3.0). Comparisons between groups were conducted using the Student's t‐test or the Wilcoxon rank‐sum test for continuous variables. For the experimental data, GraphPad Prism 8 was used to perform statistical analyses and graphics production. A value of *P* < 0.05 was considered statistically significant.

## Conflict of Interest

The authors declare no conflict of interest.

## Author Contributions

Y.X., C.C., F.W., and Y.M.P. contributed equally to this work. W.S., X.Z., and Y.X. conceived of the study. C.C., J.S., Y.Z., W.L., and Y.H. collected clinical samples. C.C. digested clinical samples and performed scRNA‐seq data analysis. H.H., Z.L., and Y.P. prepared the paraffin sections. T.X., C.C., F.W., and Y.M.P. performed IHC and mIHC staining. C.C., F.W., and Y.M.P. performed fibroblast differentiation assay and IF staining. C.C. wrote the original manuscript. W.S., X.Z., Y.X., S.P., and C.X.C. supervised the study and reviewed the manuscript. All the authors have read the final version of the manuscript and agreed to the submission.

## Supporting information



Supporting Information

## Data Availability

The data that support the findings of this study are available from the corresponding author upon reasonable request.
