# Supplementary material for: Single‐Cell Analysis Clarifies Pathological Heterogeneity in Tenosynovial Giant Cell Tumor and Identifies Biomarkers for Predicting Disease Recurrence
Source: Adv Sci (Weinh). 2025 Mar 24;12(26):2415835. doi: 10.1002/advs.202415835 (PMC12244509; doi:10.1002/advs.202415835)
Supplement: Supplementary file 1 — Supporting Information [file ADVS-12-2415835-s001.docx]

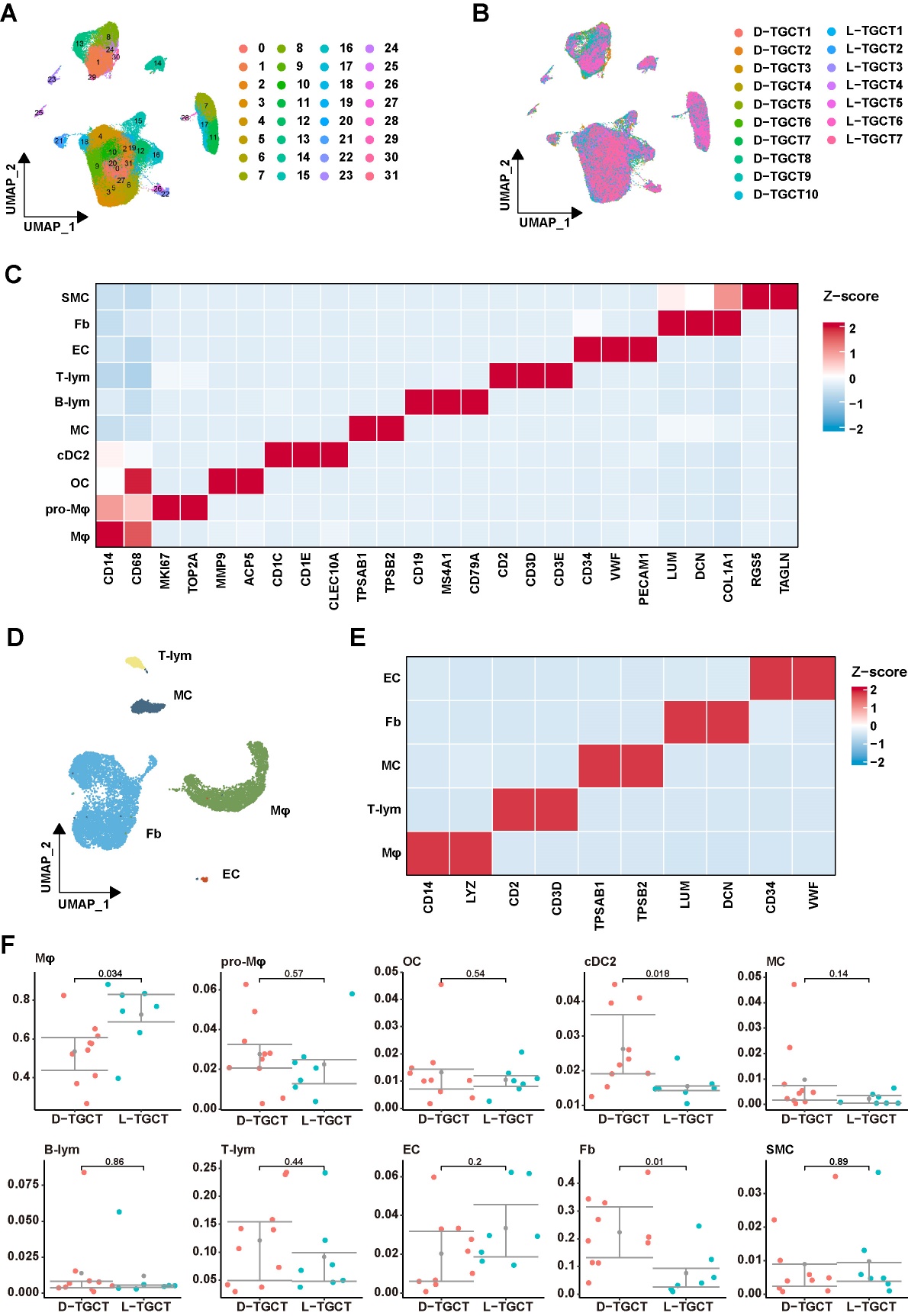
Supplementary figures

**Supplementary Figure 1.** Quality control of single-cell RNA sequencing (scRNA-seq) data. A) UMAP projection of 123,970 cells from 10 D-TGCT and 7 L-TGCT samples, which were clustered into 32 subpopulations. B) UMAP projection of all cells present in different samples. C) Heatmap of relative average expression of the selected marker genes for each cell type in TGCT. D) UMAP projection of 10,797 cells from three OA-synovium samples, which were clustered and annotated into five main cell types. E) Heatmap of relative average expression of the selected marker genes for each cell type in OA synovium. F) Box plots showing the differences in the proportions of ten main cell types between the two TGCT subtypes. Statistical significance was inferred by two-sample *t* test.

**
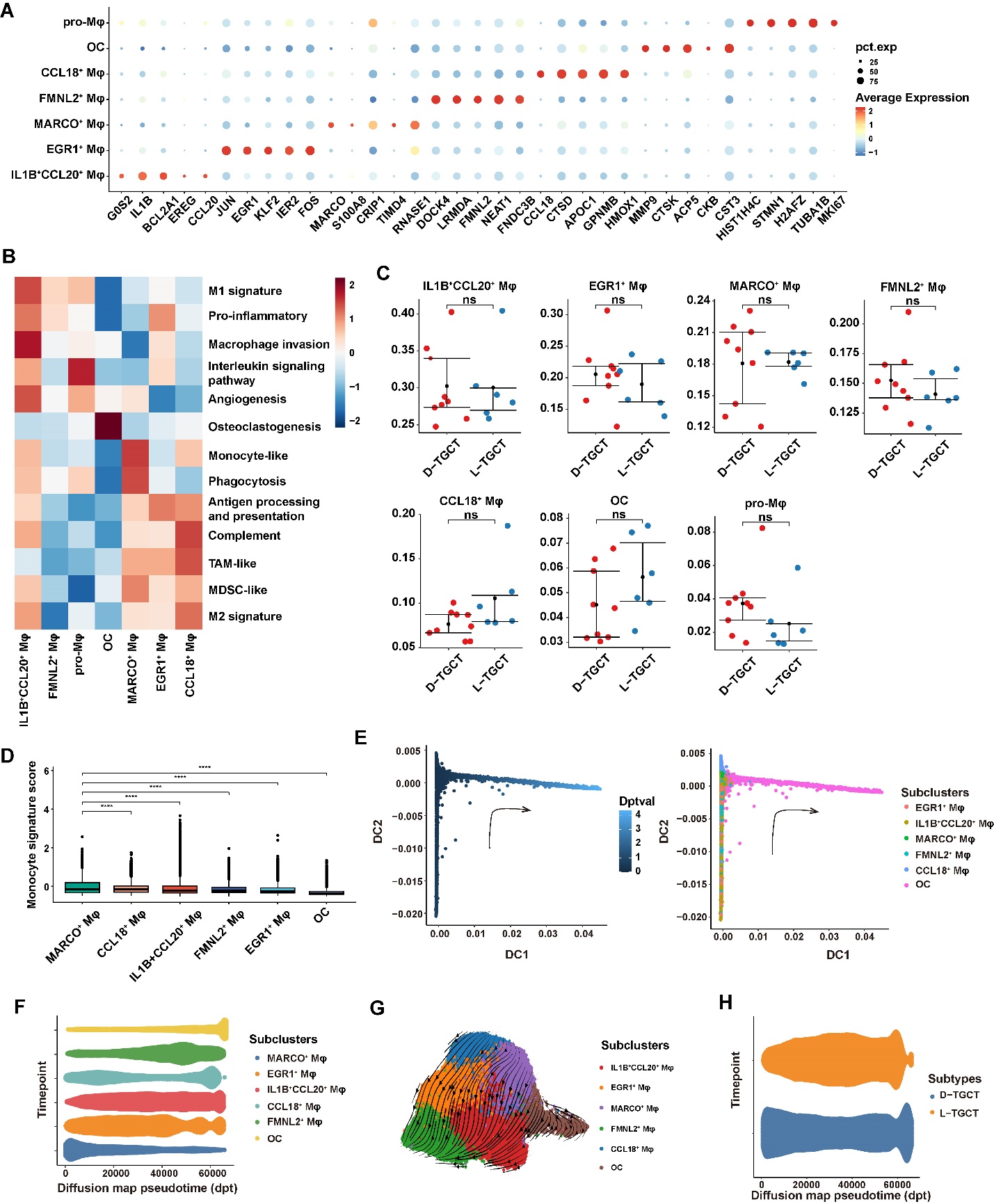
**

**Supplementary Figure 2.** Investigation of differences in proportion or functionality of macrophage subpopulations. A) Dot plot showing the expression levels of the top five DEGs of each Mφ subpopulation. B) Heatmap showing the normalized signature scores of different functional gene sets among different Mφ subpopulations. C) Box plots showing the proportions of Mφ subpopulations. Unpaired *t* test. ns = not significant. D) Box plot showing the monocyte signature score across each macrophage subpopulation. *****P* < 0.0001. E) Diffusion maps visualizing the differentiation trajectories of *IL-1B*^+^*CCL20*^+^ Mφs, *EGR1*^+^ Mφs, *MARCO*^+^ Mφs, *FMNL2*^+^ Mφs, *CCL18*^+^ Mφs, and OCs. Cells were colored by inferred diffusion pseudotime (left) and subpopulation (right). F) Macrophages were ordered by subclusters according to their diffusion map pseudotime. G) RNA velocities of *IL-1B*^+^*CCL20*^+^ Mφs, *EGR1*^+^ Mφs, *MARCO*^+^ Mφs, *FMNL2*^+^ Mφs, *CCL18*^+^ Mφs, and OCs visualized as a streamline plot. H) Macrophages were ordered by subtypes according to their diffusion map pseudotime.

**
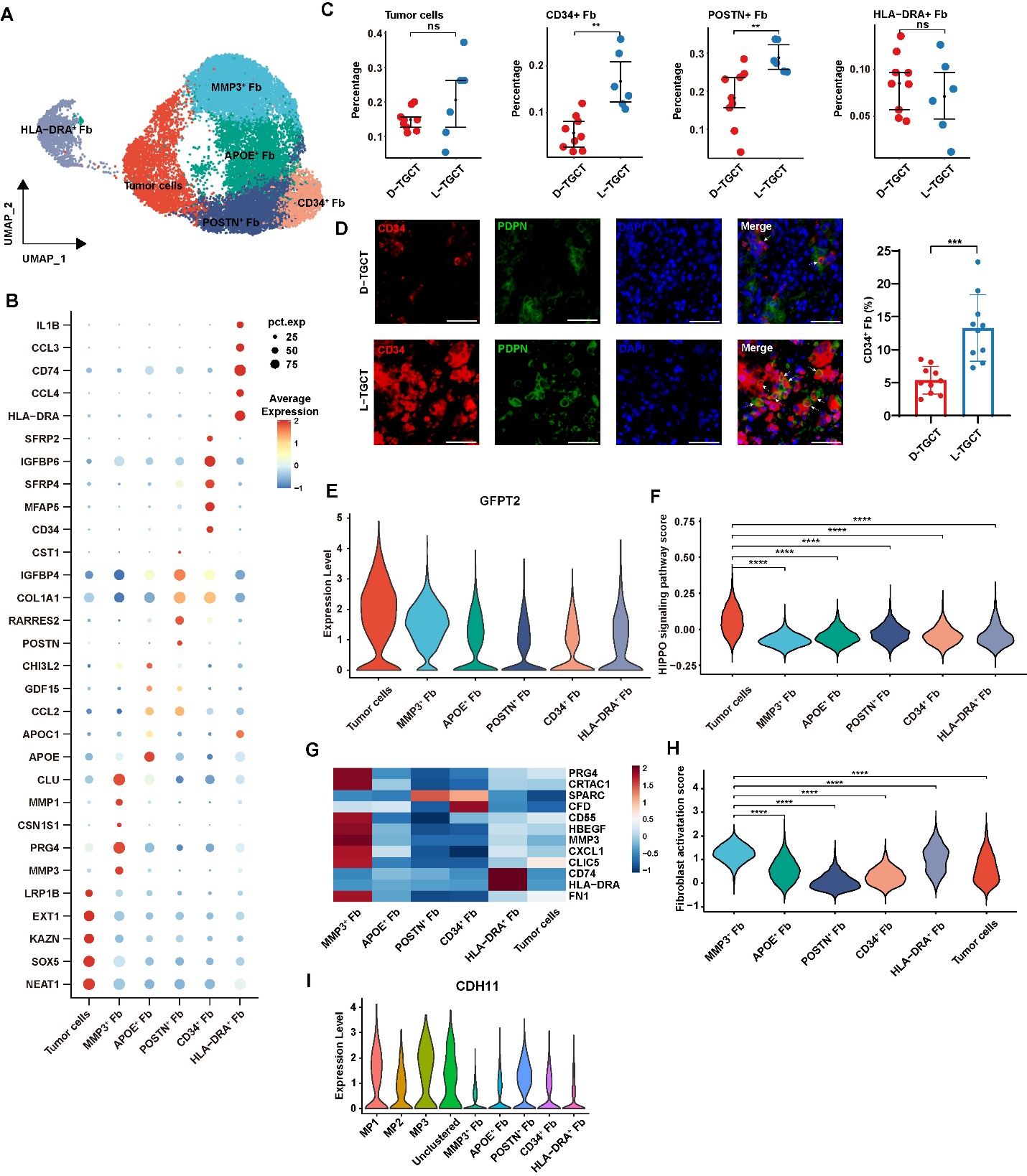
**

**Supplementary Figure 3.** Investigation of the differences in proportion or functionality of the fibroblast subpopulations. A) UMAP projection of six subpopulations generated from unsupervised clustering of Fb lineages. B) Dot plot showing the expression levels of the top five DEGs of each Fb subpopulation. C) Box plots showing the proportion differences of tumor cells, *CD34*^+^ Fbs, *POSTN*^+^ Fbs, and *HLA-DRA*^+^ Fbs between D-TGCT and L-TGCT. Unpaired *t* test. ***P* < 0.01, ns = not significant. D) Representative mIHC staining of *CD34*^+^ Fb in D-TGCT (n = 10) and L-TGCT (n = 10) lesions. Scale bar, 50 μm. Histogram on the right shows the percentages of *CD34*^+^ Fbs in D-TGCT and L-TGCT. E) Violin plot showing the expression levels of *GFPT2* across each Fb subpopulation. F) Violin plot showing the *Hippo* signaling pathway signature across each Fb subpopulation. The signature is from the Gene Ontology gene set GO:0035329. G) Heatmap showing the normalized expression levels of marker genes of an activated Fb identified in RA synovium. H) Violin plot showing the Fb activation signature across Fb subpopulations. I) Violin plot showing the expression levels of *CDH11* across Fb subpopulations.

**
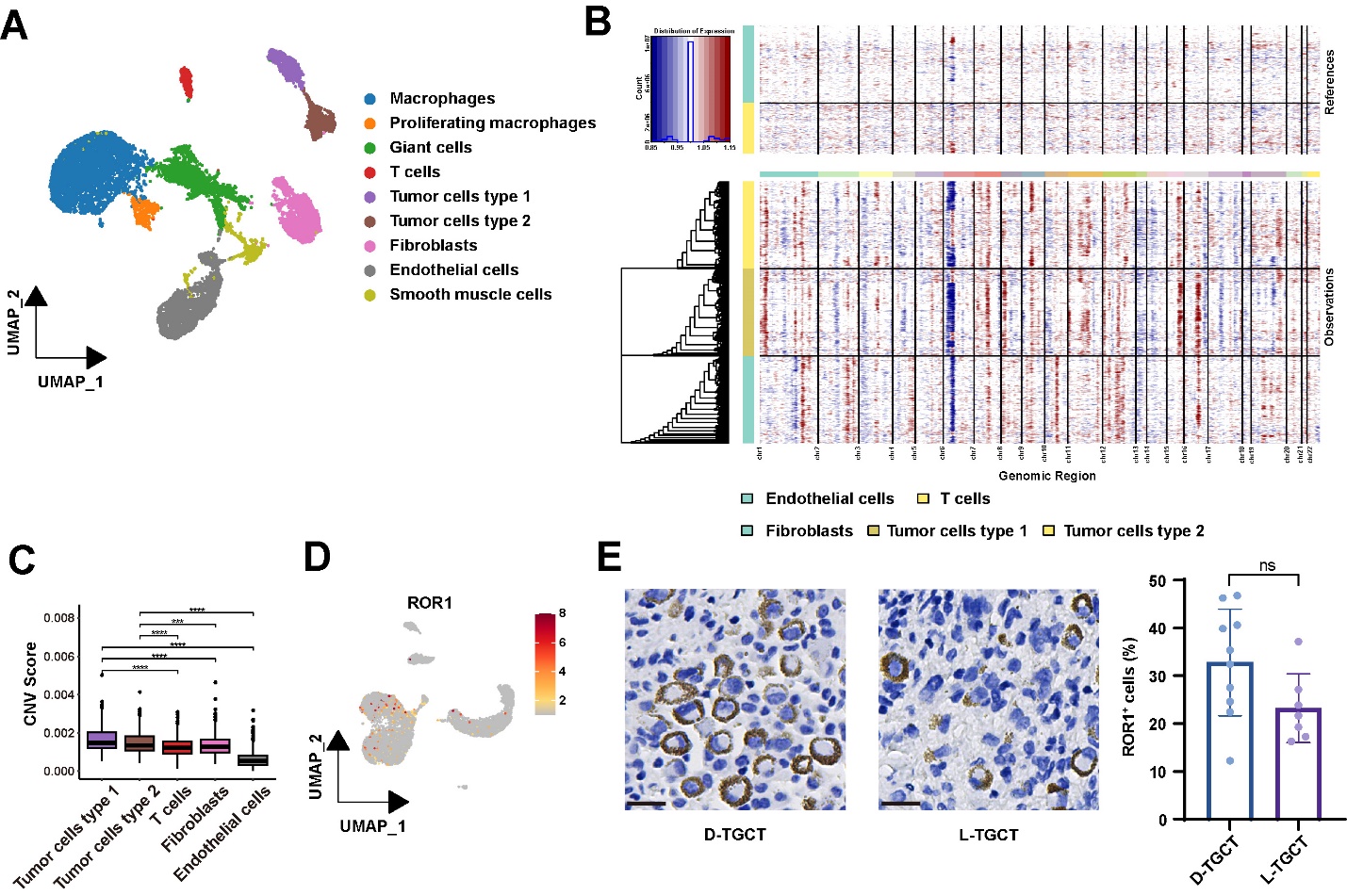
**

**Supplementary Figure 4.** CNV inference on TGCT cells from the GSE210750 dataset. A) UMAP projection of 12,077 cells from three D-TGCT samples, which were clustered and annotated into nine main cell types. B) Heatmap showing the CNV profiles of tumor cells and Fbs. ECs and T cells were selected as reference cells. Blue and red represent lost and amplified chromosomes, respectively. C) Box plot showing the CNV scores of tumor cells, Fbs, and reference cells. D) UMAP projection showing the expression levels of *ROR1* in OA synovium. Red and gray represent high and low expression levels, respectively. E) Representative IHC staining images of *ROR1* in D-TGCT (n = 10) and L-TGCT (n = 7) from discovery cohort. Scale bar, 20 μm. Histogram on the right shows the percentages of *ROR1*^+^ cells in D-TGCT and L-TGCT. Statistical significance was inferred by *t* test. ns = not significant.

**
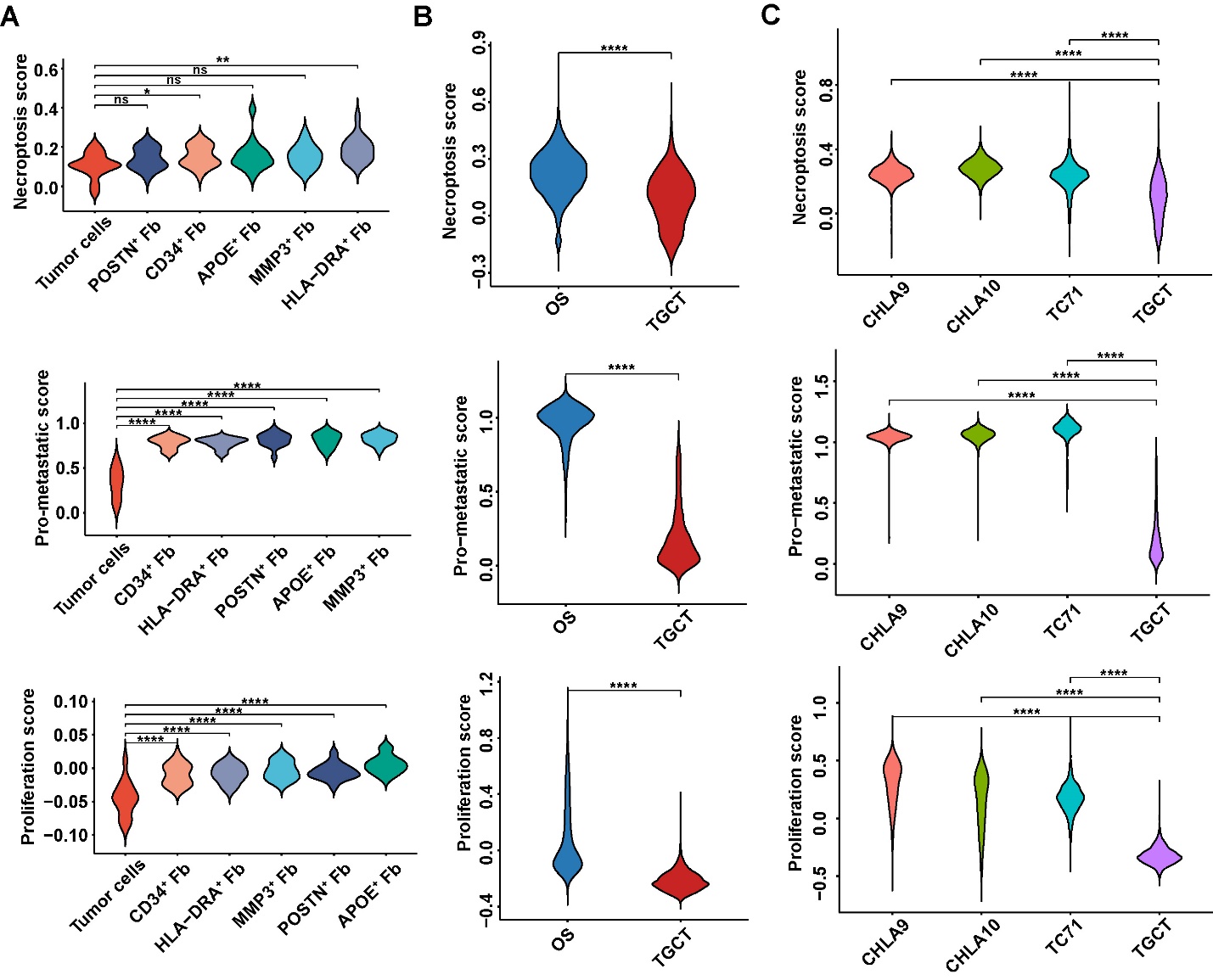
**

**Supplementary Figure 5.** Signature scores of tumor cells. A) Violin plots showing the necroptosis signature (top), pro-metastatic signature (middle), and proliferation signature (bottom) across each Fb subpopulation. B) Violin plots showing the necroptosis signature (top), pro-metastatic signature (middle), and proliferation signature (bottom) across OS tumor cells and TGCT tumor cells. C) Violin plots showing the necroptosis signature (top), pro-metastatic signature (middle), and proliferation signature (bottom) across ES cell lines and TGCT tumor cells.

**
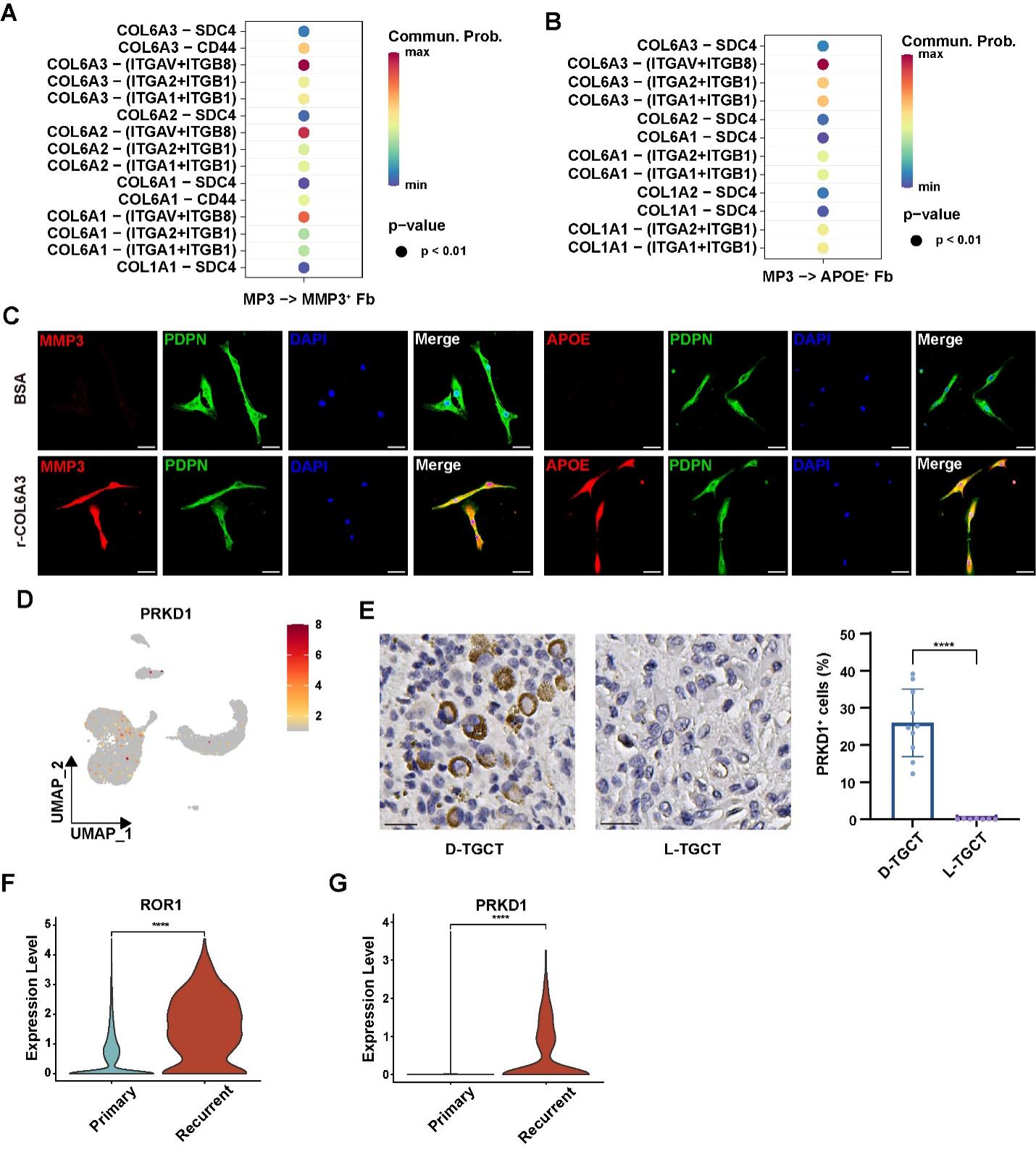
**

**Supplementary Figure 6.** The regulatory effect of tumor cells on the differentiation of fibroblasts and the expression pattern of *PRKD1*. A) Dot plot showing significant ligand–receptor interaction between MP3 and *MMP3*^+^ Fbs. B) Dot plots showing significant ligand–receptor interaction between MP3 and *APOE*^+^ Fbs. C) Representative mIHC images of *CD34*^+^ Fbs treated without (top) or with (bottom) r-COL6A3. Scale bar, 50 μm. D) UMAP projection showing the expression levels of *PRKD1* in OA synovium. Red and gray represent high and low expression levels, respectively. E) Representative IHC staining images of *PRKD1* in D-TGCT (n = 10) and L-TGCT (n = 7) from discovery cohort. Scale bar, 20 μm. Histogram on the right shows the percentages of *PRKD1*^+^ cells in D-TGCT and L-TGCT. Statistical significance was inferred by *t* test. *****P* < 0.0001. F) Violin plot showing the expression levels of *ROR1* between primary and recurrent TGCT. G) Violin plot showing the expression levels of *PRKD1* between primary and recurrent TGCT.

Supplementary tables

**Supplementary Table 1.** Clinical characteristics of 17 TGCT patients and 3 OA patients.

| Patient ID | Gender | Age | Primary/Recurrent | Site | Sequencing platform |
| --- | --- | --- | --- | --- | --- |
| D-TGCT1 | Female | 76y | Primary | Left knee | 10x Genomics |
| D-TGCT2 | Female | 29y | Recurrent | Left knee | 10x Genomics |
| D-TGCT3 | Male | 40y | Primary | Left knee | 10x Genomics |
| D-TGCT4 | Female | 45y | Primary | Left knee | 10x Genomics |
| D-TGCT5 | Female | 59y | Primary | Right knee | 10x Genomics |
| D-TGCT6 | Female | 27y | Primary | Right knee | 10x Genomics |
| D-TGCT7 | Male | 36y | Primary | Left knee | 10x Genomics |
| D-TGCT8 | Female | 59y | Primary | Left ankle | 10x Genomics |
| D-TGCT9 | Female | 58y | Primary | Right knee | 10x Genomics |
| D-TGCT10 | Female | 32y | Primary | Right knee | 10x Genomics |
| L-TGCT1 | Male | 29y | Primary | Right ring finger | 10x Genomics |
| L-TGCT2 | Female | 40y | Primary | Right thumb | 10x Genomics |
| L-TGCT3 | Female | 31y | Primary | Left middle finger | 10x Genomics |
| L-TGCT4 | Male | 37y | Recurrent | Right middle finger | 10x Genomics |
| L-TGCT5 | Male | 6y | Primary | Right middle finger | 10x Genomics |
| L-TGCT6 | Female | 41y | Primary | Left wrist | 10x Genomics |
| L-TGCT7 | Female | 36y | Primary | Right thumb | 10x Genomics |
| OA1 | Female | 69y | - | Right knee | BD Rhapsody |
| OA2 | Female | 71y | - | Left knee | BD Rhapsody |
| OA3 | Male | 66y | - | Right knee | BD Rhapsody |

**Supplementary Table 2.** Summary of public sequencing data from Gene Expression Omnibus

| Dataset | Sequencing technology | Sample |
| --- | --- | --- |
| GSE210750^29^ | scRNA-seq | GSM6436658,GSM6436659  GSM6436660,GSM6436661  GSM6436662 |
| GSE162454^39^ | scRNA-seq | GSM4952363,GSM4952364  GSM4952365,GSM5155198  GSM5155199,GSM5155200 |
| GSE146221^40^ | scRNA-seq | GSM4368462,GSM4368463  GSM4368464 |
| GSE3698^35^ | array | GSM85441,GSM85442  GSM85443,GSM85444  GSM85445,GSM85446  GSM85447,GSM85448  GSM85449,GSM85450  GSM85451 |

**Supplementary Table 3.** Signature genes used to define macrophages and fibroblasts function.

| Gene signatures | Genes |
| --- | --- |
| M1 signature^68^ | IL12, IL23, TNF, IL6, CD86, IL1B, MARCO, NOS2, CD64, CD80, CXCR10, CXCL9, CXCL10, CXCL11, IL1A, CCL5, IRF5, IRF1, CD40, IDO1, KYNU, CCR7 |
| Pro-inflammatory^68^ | IL1B, TNF, CCL2, CCL3, CCL5, CCL7, CCL8, CCL13, CCL17, CCL22 |
| Macrophage invasion^69^ | IL1B, CXCL8, TNF, TGFB1, MMP2, MMP7, MMP9, CHI3L1, SPARC |
| Interleukin signaling pathway^70^ | IL17A, IL4R, SPIC, IL9, IL15RA, MAPKAPK2, CDKN1A, STAT5A, PDPK1, RPS6KA1, STAT3, ELK3, AKT1, AKT3, IL17F, STAT4, STAT1, CXCR2, CXCR1, IRS1, IL10, ELK4, IL2RA, IL1A, IL7, PIK3CA, IL2, IL21, SHC1, NRAS, MTOR, MKNK1, NOS3, IL6, BRAF, IL5RA, RAF1, CDKN1B, IL12RB2, IL11, AKT2, MAPK3, IRS2, IKBKB, IL12RB1, JAK3, IL15, MAPK6, PIK3CB, IL10RA, IL18, IL13RA1, RPS6KA6, RPS6KA3, ARAF, ELK1, IL13RA2, FOXO3, IL20RA, MKNK2, STAT6, STAT2, IL23A, STAT5B, IL13, IL4, IL5, MAPK7, SOS2, FOS, RASA1, IL6ST, MYC, MAPK15, IL2RB, MAPK1, GSK3B, IL10RB, RPS6KA2, SOS1, SRF, CHUK, IL6R, SPI1, IL11RA, CXCL8 |
| Angiogenesis^70^ | PRG2, JAG2, COL5A2, COL3A1, VAV2, ITGAV, JAG1, THBD, POSTN, S100A4, PDGFA, LRPAP1, MSX1, PF4, SPP1, CCND2, OLR1, KCNJ8, PGLYRP1, NRP1, FGFR1, LPL, SLCO2A1, TIMP1, LUM, APOH, VTN, SERPINA5, VCAN, STC1, PTK2, APP, FSTL1, TNFRSF21, VEGFA, CXCL6 |
| Osteoclastogenesis^71^ | FOS, JDP2, SPNS2, CTSK, EFNB2, E2F1, FOXM1, OSTM1, ITGB3, CKB, ACP5, ATP6V0D2, NFATC1, CD109, OSCAR, CA2, CALCR, MMP9, CTHRC1, PPARGC1B, MITF |
| Mono-like^72^ | S100A8, S100A9, S100A12, VCAN, CSF3R |
| Phagocytosis^70^ | RPS6KB1, RAC2, PRKCD, PRKCB, SYK, VASP, INPPL1, AKT1, GSN, INPP5D, PTPRC, PLA2G4A, AKT3, ARPC2, ARPC5, FCGR2B, VAV2, PLA2G4E, HCK, PLCG1, ARPC5L, PLA2G4B, PLA2G4D, PLA2G4F, PLD1, PIP5K1A, VAV3, PIK3CA, LYN, ASAP3, PIK3CD, PIK3R3, MARCKSL1, WASF2, CDC42, LIMK1, ARPC1B, ARPC3, NCF1, RAC1, ARPC1A, WASF3, RAF1, WASL, ARPC4, GAB2, AKT2, SPHK2, PAK1, LAT, MAPK3, PLCG2, PIK3R2, DNM2, MAP2K1, PIK3CB, WAS, WASF1, MARCKS, PIP5K1C, CRK, PRKCA, LIMK2, DOCK2, PLD2, SPHK1, ASAP2, SCIN, CFL2, ARF6, AMPH, PIK3R1, ASAP1, MYO10, PLA2G6, MAPK1, CRKL, PRKCE, VAV1, BIN1, PIP5K1B, RPS6KB2, CFL1, PRKCG, FCGR2A, FCGR1A, IGH, FCGR3A, PLPP1, PLPP3, PLPP2 |
| Antigen processing and presentation^70^ | CTSB, CREB1, PDIA3, HSPA5, B2M, RFX5, RFXAP, CTSS, NFYC, CD8A, CD4, KLRC3, KLRC1, KLRC2, KLRD1, RFXANK, IFI30, CALR, HSPA8, KIR3DL2, KIR3DL1, NFYB, IFNG, HSPA4, CANX, PSME3, HSPA2, LGMN, HSP90AA1, CTSL, PSME2, PSME1, CIITA, TAPBP, TAP1, TAP2, HSPA1A, HSPA1B, HSPA1L, TNF, HSP90AB1, NFYA, CD74, KLRC4, HLA-DOA, HLA-F, KIR2DL1, KIR2DL3, KIR2DS4, HLA-DQA1, HLA-DMB, HLA-DRA, HLA-DPB1, HSPA6, HLA-DMA, HLA-DPA1, HLA-G, HLA-A, HLA-DRB5, HLA-E, HLA-DOB, HLA-B, CD8B, HLA-C, KIR2DL4, KIR2DS1, KIR2DS3, KIR2DS5, HLA-DQB1, KIR2DL5A, KIR2DS2, KIR2DL2, HLA-DQA2, HLA-DRB1, KIR3DL3, HLA-DRB3, HLA-DRB4 |
| Complement^70^ | CD55, SERPINE1, MMP15, EHD1, KLK1, GZMB, PRKCD, PIK3R5, GNAI2, TIMP2, SH2B3, SRC, CTSB, CASP10, DOCK10, FN1, PLA2G4A, SERPINC1, CR2, CD46, VCPIP1, SERPINB2, RABIF, CSRP1, CFH, F5, FCER1G, KYNU, GPD2, GCA, GATA3, HSPA5, DPP4, SERPING1, F2, RASGRP1, USP8, ADRA2B, KCNIP3, HNF4A, CEBPB, GNB4, CP, PIK3CA, ANXA5, CTSO, S100A13, S100A9, CTSS, GNAI3, F3, LYN, LCK, C1QC, PDP1, PCSK9, HPCAL4, C1QA, CDA, CASP9, PCLO, CD36, IL6, RNF4, LAP3, CXCL1, GNB2, RAF1, TFPI2, PRSS3, GP9, C1S, OLR1, CALM3, APOC1, PLAUR, CTSC, PRCP, RHOG, PPP4C, PRSS36, ITGAM, IRF7, CTSD, CDH13, COL4A2, ADAM9, IRF2, CASP3, F10, F7, PLAT, PPP2CB, KLKB1, MT3, SCG3, ME1, CASP1, CASP4, MMP13, MMP12, MMP8, APOA4, FDX1, CTSH, PLSCR1, LTF, LAMP2, F8, PHEX, WAS, TIMP1, CD40LG, FYN, PREP, LTA4H, USP15, LRP1, TNFAIP3, SPOCK2, SIRT6, PRDM4, DUSP6, CPM, DYRK2, PLEK, LCP2, AKAP10, RF1, ATOX1, GP1BA, PFN1, CDK5R1, CCL5, GNGT2, GRB2, DOCK4, PSEN1, PIK3C, CALM1, LGMN, ACTN2, CTSL, KIF2A, GZMA, GZMK, GNG2, DGKH, DOCK9, ITIH1, GMFB, LGALS3, ANG, MMP14, CLU, ZFPM2, C9, TMPRSS6, MAFF, PDGFB, DGKG, CBLB, USP16, PLG, BRPF3, CFB, L3MBTL4, PIM1, PSMB9, NOTCH4, HSPA1A, C2, PLA2G7, C3, ZEB1, USP14, JAK2, KCNIP2, XPNPEP1, DUSP5, CASP7, RCE1, LIPA, CD59, CASP5, SERPINA1, STX4, CA2, APOBEC3G, CPQ, ERAP2, CR1, FCN1, APOBEC3F, C4BPB, C1R, S100A12, MSRB1, CTSV, RBSN |
| TAM-like^72^ | APOE, C1QA, C1QB, TREM2, SLC40A1, GPNMB, CST3, LYZ, CD68, CD163, CD169, ID3, MITF, RUNX2, MAF |
| MDSC-like^73^ | CST3, LYZ, CD68, THBS1, S100A8, S100A9, BCL3, NR4A1, RXRA, TCF25 |
| M2 signature^68^ | ARG1, ARG2, IL10, CD32, CD163, CD23, CD200R1, PDCD1LG2, CD274, MARCO, CSF1R, MRC1, IL1RN, IL1R2, IL4R, CCL4, CCL13, CCL20, CCL17, CCL18, CCL22, CCL24, LYVE1, VEGFA, VEGFB, VEGFC, VEGFD, EGF, CTSA, CTSB, CSTC, CTSD, TGFB1, TGFB2, TGFB3, MMP14, MMP19, MMP9, CLEC7A, WNT7B, FASL, TNFSF12, TNFSF8, CD276, VTCN1, MSR1, FN1, IRF4 |
| Necroptosis^74^ | AIFM1, ATG9A, ATG9B, BNIP3, CASP6, HEBP2, MIR103A1, MIR107, MIR92A1, MT-CO2, MT3, MTCO2P12, PARP1, RIPK1, RIPK3, SLC6A13, SLC6A6, TP53, TSPO, ZBP1 |
| Pro-metastatic^74^ | WSB1, KARS, TUBB2A, DSTN, PSMB1, MRPS23, CCT8, SPCS1, PAPOLA, POLR2C, MYL12A, PFDN4, ARPC1A, DNAJB11, EIF4B, EMG1, ESF1, TRAPPC1, NOP58, KNOP1, DDOST, FAM3B, SF3B4, ADSL, PTGDS, MRPL28, DRG1, ATP6V1F, MLF2, RP11-242D8.1, CREB5, BUB3, ANP32A, ODC1, NASP, RSL24D1, BST2, CCT4, HNRNPA0, SERPINB1, SET, RP11-402P6.11, CD63, MCM3, LINC00493, EIF4A1, PSMA4, CCT5, POMP, PRSS23, CHMP5, ANP32B, EEF1G, SNHG8, MAGEA1, HNRNPK, HBA2, UBE2L3, ATIC, PPIC, PCSK1N, MARCKS, PGAM2, YWHAE, COPE, EEF1D, CNN2, LGALS2, SNRPG, MT1F, SELK, KCNQ1OT1, GDF15, POLR2H, CCNB1, OSTC, HSPE1, ARL6IP1, MLF1, C4orf48, MRPL3, MYL6, ROMO1, RPS4Y1, APEX1, GSTM3, PPA1, GGH, GTF3C6, SFT2D1, FKBP10, TOP2A, TXN, HNRNPA3, SERBP1, RPL9, RPL39L, PRMT1, PKIB, EIF4A2, MEST, RSU1, RAB11A, ACTL8, CHCHD2, DSCR8, GTPBP4, MORF4L1, DCTPP1, BRIX1, DDX21, CNOT10, PRSS3, TPM4, C14orf2, IFITM2, AKR1C3, KRT18, AHSA1, SLIRP, MT1H, RPA3, FKBP4, GNL3, IFI27L2, CCDC124, S100A13, HIST1H4C, CALM1, MRPL51, EIF1B, TSC22D1, CALR, MYL9, CDC20, RPS16, CCT3, HSPD1, GGCT, NOP56, FKBP1A, LSM2, RBM3, BZW2, GMNN, LINC00152, IER3, ACP1, AKR1C1, RPL22, CDC123, ERGIC3, SAP18, AKR1C2, PSMD7, SSB, ZNRD1, KDELR1, RPS20, C19orf33, CLDN4, KRT17, SEC11A, ATXN10, ABRACL, DDAH2, SF3B6, SNRPD3, TCEB2, CCT2, LY6E, BIRC5, GLUL, NACA, KRT10, MAPRE1, CPNE1, CYR61, TSPO, HNRNPC, MRPL13, RBM8A, C14orf166, SNU13, MRPS35, PFDN5, PPP4C, TMEM258, MAGEC2, HSPH1, RAC1, SERPINH1, GDI2, ZFAS1, MCM7, PLIN2, RBX1, AP2M1, CYCS, RPS25, HDAC2, TCP1, HERPUD1, ENY2, XRCC6, PTTG1, FUS, CSNK2B, REG1A, RPL27A, RPS7, LAPTM4B, UQCRB, CFL1, SEC61G, CD9, AHCY, SNRPF, EIF3M, PHLDA2, EIF6, CDK4, MRPL18, POLR2K, PRDX2, PRAME, FXYD2, POLR2F, SUMO2, EIF1, EIF3L, MEG3, PAFAH1B3, LEFTY1, SEPW1, RPL11, LYRM5, MYC, HSPA8, CCT6A, TSPAN8, H3F3B, BTG2, HNRNPDL, TPM2, TKTL1, ILF2, APRT, FAM96B, GP2, KPNA2, SLC25A6, PA2G4, C19orf53, H3F3A, RSL1D1, RP11-14N7.2, CRTAP, FBL, HSPB1, PDCD6IP, ITM2A, CKLF, DYNLL1, KRAS, GTSF1, ALDH3A1, PTGR1, LY6K, SNRPD2, ANXA5, TMN1, BTF3, UBE2I, PGAM1, PTMA, TUBB4B, TMA7, HSPA5, RNPS1, HMGN2, LGALS3, CHI3L1, CLDN6, PLA2G2A, HNRNPA2B1, DCAF13, KRT8, PCNACRIP1, CBX3, EEF1B2, GAGE13, PLP2, SQSTM1, RPL37, RPS26, LSM5, ATP5G2, PAGE2, SNRPE, CXCL1, SRSF3, XIST, RPS27A, CCL20, SNRPD1, NUTF2, TUBB2B, ANXA1, COMMD6, TPT1, RPLP0, LSM7, NAP1L1, TPI1, CD24, ANXA4, TOMM22, RPL10, RPL13A, EIF3D, RPS29, NBEAL1, RPS27, PHB, SNRPB2, WFDC2, EMP3, RPL6, ADIRF, DNAJA1, CXCL3, RPL19, HES1, CXCL8, DMKN, RPL27, KRT19, HMGB1, CKS2, EIF3H, DNAJB1, RPLP2, RPL23A, RPL17, RPS12, RPL30, GAPDH, G6PD, STOML2, IMPDH2, RPS9, UBA52, RPL41, RPL14, RPS11, ERH, YWHAZ, EEF2, EPCAM, RPL29, MARCKSL1, FBLN1, RPS13, VIM, HSPA1B, GNB2L1, RPS8, RAN, RPL24, LGALS3BP, S100A10, HSPA1A, TMSB4X, KIAA0101, SH3BGRL3, RPSA, RPL22L1, AC090498.1, RPL21, UCHL1, ELF3, RPS28, RPL13, SNHG25, PPIA, RPL3, HNRNPA1, RPS5, RPL7A, TAGLN2, EEF1A1, BEX1, RPL8, RPS4X, RPL18, RPS14, HMGB2, RPL4, TUBA1C, RPS24, RPL23, PAGE2B, TOMM7, RPL35, RPL28, EIF3E, RPL5, VCX2, HN1, PAGE1, RPL26, RPL34, UBE2C, RPL37A, RPL15, PABPC1, RPL10A, S100A9, RPS3A, RPS15A, RPL32, HSBP1, HSPA6, DEFB1, H2AFZ, ACTB, RPL31, ZFAND2A, GAGE12H, CYBA, TKT, RPL38, RPS15, ACTG1, SNRPB, HSP90AB1, RPL7, S100P, BSG, CKB, GLB1, NDUFA4L2, SPINK1, RANBP1, RPL35A, HSP90AA1, RPL36, RPL39, GSTP1, UBB, VCX, MIF, RPS19, RPS23, NPM1, RPL12, TFF1, RPS18, FABP5, LGALS1, RPS21, ANXA2, RPS17, PPDPF, RPS3, TUBA1B, GAGE2A, PKM, HMGA1, TMSB10, RPL36A, RPL18A, TM4SF1, TIMP1, CLIC1, IGHG3, RPS2, LDHB, ALDOA, NQO1, GAGE1, TFF3, MDK, GPX2, PAGE5, RPS6, TFF2, S100A11, VCX3A, SPP1, S100A6, AGR2, LYZ |
| Proliferation^74^ | ZWINT, E2F1, FEN1, FOXM1, H2AFZ, HMGB2, MCM2, MCM3, MCM4, MCM5, MCM6, MKI67, MYBL2, PCNA, PLK1, CCND1, AURKA, BUB1, TOP2A, TYMS, DEK, CCNB1, CCNE1 |
| Fibroblast activation^31^ | PRG4, CRTAC1, SPARC, CFD, CD55, HBEGF, MMP3, CXCL1, CLIC5, CD74, HLA-DRA, FN1 |

**Supplementary Table 4.** Strength of correlative CCI network referring to Figure 4D.

| Sender | Receiver | Strength |
| --- | --- | --- |
| Tumor cells | *CD34*^+^ Fbs | 0.114657392 |
| Tumor cells | *MMP3*^+^ Fbs | 0.078661305 |
| Tumor cells | *APOE*^+^ Fbs | 0.067088816 |
| Tumor cells | *POSTN*^+^ Fbs | 0.053389764 |
| Tumor cells | *HLA-DRA*^+^ Fbs | 0.014302769 |

**Supplementary Table 5.** Strength of correlative CCI network referring to Figure 5D.

| Sender | Receiver | Strength |
| --- | --- | --- |
| MP3 tumor cells | *MMP3*^+^ Fbs | 0.028292316 |
| *POSTN*^+^ Fbs | *MMP3*^+^ Fbs | 0.021671807 |
| Smooth-muscle cells | *MMP3*^+^ Fbs | 0.019526514 |
| Epithelial cells | *MMP3*^+^ Fbs | 0.011338354 |
| *CD34*^+^ Fbs | *MMP3*^+^ Fbs | 0.010753132 |
| T lymphocytes | *MMP3*^+^ Fbs | 0.004404962 |
| *APOE*^+^ Fbs | *MMP3*^+^ Fbs | 0.003650853 |
| MP3 tumor cells | *APOE*^+^ Fbs | 0.005844452 |
| Epithelial cells | *APOE* ^+^ Fbs | 0.005731708 |
| MP1 tumor cells | *APOE*^+^ Fbs | 0.002473993 |
